# Evaluation of the genotoxicity of cell phone radiofrequency radiation in male and female rats and mice following subchronic exposure

**DOI:** 10.1002/em.22343

**Published:** 2019-11-13

**Authors:** Stephanie L. Smith‐Roe, Michael E. Wyde, Matthew D. Stout, John W. Winters, Cheryl A. Hobbs, Kim G. Shepard, Amanda S. Green, Grace E. Kissling, Keith R. Shockley, Raymond R. Tice, John R. Bucher, Kristine L. Witt

**Affiliations:** ^1^ Division of the National Toxicology Program National Institute of Environmental Health Sciences Research Triangle Park North Carolina; ^2^ Integrated Laboratory Systems, Inc. Research Triangle Park North Carolina; ^3^ Biostatistics and Computational Biology Branch, National Institute of Environmental Health Sciences Research Triangle Park North Carolina

**Keywords:** DNA damage, micronucleus assay, comet assay, brain, Sprague Dawley, glioma

## Abstract

The National Toxicology Program tested two common radiofrequency radiation (RFR) modulations emitted by cellular telephones in a 2‐year rodent cancer bioassay that included interim assessments of additional animals for genotoxicity endpoints. Male and female Hsd:Sprague Dawley SD rats and B6C3F1/N mice were exposed from Gestation day 5 or Postnatal day 35, respectively, to code division multiple access (CDMA) or global system for mobile modulations over 18 hr/day, at 10‐min intervals, in reverberation chambers at specific absorption rates of 1.5, 3, or 6 W/kg (rats, 900 MHz) or 2.5, 5, or 10 W/kg (mice, 1,900 MHz). After 19 (rats) or 14 (mice) weeks of exposure, animals were examined for evidence of RFR‐associated genotoxicity using two different measures. Using the alkaline (pH > 13) comet assay, DNA damage was assessed in cells from three brain regions, liver cells, and peripheral blood leukocytes; using the micronucleus assay, chromosomal damage was assessed in immature and mature peripheral blood erythrocytes. Results of the comet assay showed significant increases in DNA damage in the frontal cortex of male mice (both modulations), leukocytes of female mice (CDMA only), and hippocampus of male rats (CDMA only). Increases in DNA damage judged to be equivocal were observed in several other tissues of rats and mice. No significant increases in micronucleated red blood cells were observed in rats or mice. In conclusion, these results suggest that exposure to RFR is associated with an increase in DNA damage. Environ. Mol. Mutagen. 61:276–290, 2020. © 2019 Wiley Periodicals, Inc.

## INTRODUCTION

Over the past two decades, cellular telephone use has become nearly ubiquitous worldwide; cell phone subscriptions numbered ~7.68 billion in 2017 according to the International Telecommunication Union (2017) with ~5.12 billion unique subscribers (GSMA Intelligence 2019). Radiofrequency radiation (RFR) is a form of electromagnetic radiation that ranges from 3 kHz to 300 GHz. Most cell phones transmit RFR signals within the 800–900 and 1,800–2,200 MHz ranges (International Agency for Research on Cancer [IARC] Working Group on the Evaluation of Carcinogenic Risks to Humans 2013).

Concern exists as to whether cell phone RFR frequencies are capable of adversely affecting human health. Although some epidemiological studies suggest that cell phone use might increase the risk for certain brain cancers, such as gliomas and acoustic neuromas (a,k,a, vestibular schwannomas), the odds ratios for these increased risks are quite low (INTERPHONE Study Group 2010; Cardis et al. 2011; Hardell et al. 2011; Larjavaara et al. 2011; Sato et al. 2011; Hardell and Carlberg 2015). Conclusions drawn from these observations may be premature, as cell phone use has become commonplace only within the past two decades, a period of time that may be insufficient to accurately assess cancer‐related outcomes. Results of previous rodent cancer studies conducted with a variety of RFR exposures and durations are inconsistent and inconclusive, and many of these studies used experimental protocols with important limitations, indicating a need for a more definitive study (IARC Working Group on the Evaluation of Carcinogenic Risks to Humans 2013).

Additionally, extensive reviews of the literature on the genotoxicity of various frequencies and modulations of RFR have concluded that evidence for RFR‐associated genotoxicity is inconsistent and weak (Brusick et al. 1998; Ruediger 2009; Verschaeve et al. 2010), and some key studies reporting RFR‐associated genotoxicity in human cell lines could not be replicated (Speit et al. 2013). As with the cancer studies, interpretations of the genotoxicity studies, particularly those performed *in vivo*, have also been limited by issues of experimental design. In 2013, after reviewing the available data, the IARC classified radiofrequency electromagnetic fields (RF‐EMF), which include the RFR wavelength range, as “possibly carcinogenic to humans (Group 2B),” based on limited evidence in experimental animals and limited evidence in humans on the association between RF‐EMF and cancer (IARC Working Group on the Evaluation of Carcinogenic Risks to Humans 2013).

To help inform human health risk assessments, the National Toxicology Program (NTP) designed and conducted a 2‐year rodent cancer study of cell phone RFR, using code division multiple access (CDMA) or global system for mobile (GSM) modulations, the principal modulations used in the United States (CDMA and GSM) and in the rest of the world (GSM). GSM and CDMA are second‐generation (2G) and third‐generation (3G) technologies, respectively, and they differ in the method in which information is incorporated and transmitted within frequency bands. The previous inconsistent genotoxicity and tumorigenicity findings that have been reported following RFR exposure could be due in part to the immense and unique technical challenges inherent in studying the effects of nonionizing radiation, including RFR (Capstick et al. 2017; Gong et al. 2017). To address these challenges and provide data to clarify possible adverse biological effects of cell phone RFR exposure, the NTP took into account numerous variables and parameters in designing its rodent cancer bioassay. Key features included construction of custom‐designed reverberation chambers that exposed animals to a clearly defined, statistically homogenous radiofrequency field, that shielded animals from all other sources of RFR, and eliminated the need for restraint, a method commonly employed by other researchers for point‐source exposures (Capstick et al. 2017; Gong et al. 2017). Animals were housed inside the reverberation chambers and exposed to RFR for a total of 9 hr 10 min per day in 10‐min on/off cycles (over the course of an ~18 hr period) at frequencies with modulations being used in cellular networks (Capstick et al. 2017). In addition, the exposure levels selected for this study were based on the results of previously conducted dosimetry studies and thermal pilot studies that demonstrated no measurable hyperthermia in rats and mice at the exposure levels chosen for this study (Gong et al. 2017; Wyde et al. 2018).

In the NTP study design, Sprague Dawley rats and B6C3F1/N mice of both sexes were whole‐body exposed to RFR (CDMA or GSM modulations). Rats were exposed *in utero* beginning on Gestation day 5 (GD5), and mice were exposed beginning at 5 weeks of age. After a total of 19 weeks of exposure for rats and 14 weeks for mice, subsets of 5 rats and 5 mice of each sex from each exposure group were removed from the ongoing 2‐year cancer bioassay after subchronic exposure and assessed for DNA damage using the comet assay, and for changes in chromosomal structure and/or number using the peripheral blood erythrocyte micronuclei (MN) assay. For the comet assay, cells from three functionally distinct structures of the brain (frontal cortex, hippocampus, and cerebellum), along with liver cells and peripheral blood leukocytes were assessed. Brain tissue was analyzed in the comet assay due to concerns that RFR may increase risk for brain cancer in humans, whereas liver cells and blood leukocytes were selected for analysis as these cells are part of typical analyses conducted at the NTP for DNA damage.

## MATERIALS AND METHODS

### Animal Husbandry

Time‐mated Hsd:Sprague Dawley SD rats (11–14 weeks of age) (Harlan, Indianapolis, IN) were received on GD2 at the laboratory (Illinois Institute of Technology Research Institute; IITRI, Chicago, IL). After littering, male and female pups were housed with their dams until weaning on Postnatal day 28 (PND28). During the perinatal phase, rats were fed irradiated NIH‐07 wafers; from weaning until study completion, rats were fed irradiated NTP‐2000 rodent diet (Zeigler Brothers, Gardners, PA). Male and female B6C3F1/N mice (Taconic, Germantown, NY) were received at 3–4 weeks of age. Mice were quarantined for 10–14 days and were 5–6 weeks of age at the start of exposure. Mice were fed irradiated NTP‐2000 rodent diet. All animals were provided food and tap water (city of Chicago, IL, municipal supply) *ad libitum*. During the studies, animal health was monitored according to the NTP sentinel animal program. Mice, and rats after weaning, were housed individually in solid polycarbonate cages with irradiated hardwood bedding (Sani‐chips, P.J. Murphy, Montville, NJ) within custom designed, stainless steel reverberation chambers. Environmental conditions were set to maintain a 12‐hr light/dark cycle, a temperature of 72 ± 3°F, a humidity range of 50 ± 15%, and >10 air changes/hr.

Animal use was in accordance with the U.S. Public Health Service policy on humane care and use of laboratory animals and the Guide for the Care and Use of Laboratory Animals (National Research Council 1996). Animal housing facilities were accredited by the Association for Assessment and Accreditation of Laboratory Animal Care; all procedures were approved by the IITRI Institutional Animal Care and Use Committee. The RFR exposures performed at IITRI were in compliance with Food and Drug Administration Good Laboratory Practice Regulations (21CFR, Part 58). Animals were euthanized by CO_2_ asphyxiation.

### Reverberation Chambers

Reverberation chambers were self‐contained rooms that were designed to house unrestrained animals in cages and expose them to a uniform field of RFR (GSM or CDMA) and to shield animals from all outside RFR. Detailed descriptions of the design of the reverberation chambers and the RFR exposure system are provided in Capstick et al. (2017) and Gong et al. (2017). Uniformity of the RFR field was achieved by installing excitation antennas with rotating horizontal and vertical reflective surface paddles to ensure uniform distribution of statistically homogenous RFR fields within the volume of the chambers. Therefore, animals were exposed to all polarizations of RFR fields from all directions regardless of their posture or orientation to the antenna. Animals were housed one per cage to prevent interference in energy absorption. Cages, cage racks, and materials used to deliver food and water to the animals were designed to minimize interference with RFR exposure. Chamber design and animal housing were developed in collaboration with the National Institute of Standards and Technology (NIST) and the Foundation for Research on Information Technologies in Society (IT'IS). RFR field intensity, uniformity, quality of modulation, and numerous other parameters were validated by NIST, and consistency of exposure was monitored in real time by IT'IS. Further evaluation of the exposure systems is presented in NTP Technical Reports 595 (NTP 2018a) and 596 (NTP 2018b).

### Dosimetry, Specific Absorption Rates, and Exposure Regimen

Briefly, in pilot studies, body temperatures were monitored using subcutaneously implanted temperature chips (Wyde et al. 2018). Both young and older animals were tested for the possibility of thermal effects from radiation. An upper limit of 1°C was set as an acceptable increase in body temperature. Models predicted that thermoregulation might not be maintained in rats exposed to an specific absorption rate (SAR) > 6.0 W/kg, delivered at a frequency of 900 MHz, and in mice exposed to an SAR > 10.0 W/kg, delivered at a frequency of 1,900 MHz (Gong et al. 2017; Wyde et al. 2018). Thus, these were selected as the highest exposure levels in the current study, and the two lower exposures were each reduced by half. Due to technical constraints, body temperatures could not be monitored in the current study.

Rats were exposed to SARs of 0, 1.5, 3.0, or 6.0 W/kg (CDMA or GSM) RFR (900 MHz) beginning *in utero* at GD5 and continuing through gestation (~2 weeks) until weaning at PND28. Exposures continued for 14 weeks after weaning. Mice were exposed to SARs of 0, 2.5, 5.0, or 10.0 W/kg (CDMA or GSM) RFR (1,900 MHz) for 14 weeks beginning at 5–6 weeks of age. Rat exposures were initiated at the time of implantation (GD5) to simulate whole‐life exposures in humans, but because B6C3F1/N mice are poor and unpredictable breeders, this animal model is not suitable for whole‐life exposure assessments. Exposures ran daily from 11:00 a.m. to 2:00 p.m. and from 3:40 p.m. to 7:00 a.m., with RFR cycling on and off every 10 min, resulting in a total duration of exposure of 9 hr 10 min per 24‐hr period. This exposure schedule accommodated two daily intervals for animal care. Animals were housed individually in a total of 21 reverberation chambers, 7 for the mice and 14 for the rats. Each reverberation chamber emitted one power level for one modulation. Male and female mice, due to similarity in weight and size, were exposed together in 7 reverberation chambers. In contrast, due to gender‐related differences in weight and size, male and female rats were exposed in separate chambers, thus requiring 14 chambers. To control for possible positional differences in RFR field strength, cages were rotated in the racks weekly. Because SAR is dependent on body weight, the energy used to emit RFR was adjusted twice weekly for rats and once weekly for mice based on the average weight of all animals in an exposure chamber.

The sham control rats and mice were housed in reverberation chambers without activation of RFR. One group of five animals of each sex/species served as the sham control for both CDMA and GSM exposures.

### Tissue Sample Collection

On the day of necropsy, RFR exposure ceased at 7 a.m. Necropsies were performed in two shifts. For each species, 35 male animals (5 controls, 15 exposed to CDMA, and 15 exposed to GSM) were necropsied 1.5–4 hr after cessation of exposure and 35 female animals (5 controls, 15 exposed to CDMA, and 15 exposed to GSM) were necropsied approximately 4.5–7 hr after cessation of exposure. Animals were necropsied in the following order: one animal from each dose group starting with the sham exposed group, moving through each dose group for each RFR modulation in turn, then rotating back to the sham control group; animals were necropsied in numerical order within each dose group. Five tissues were collected from each animal for the comet assay. One blood sample per animal collected by retro‐orbital bleeding was divided into two aliquots: one for the comet assay and the other for the MN assay.

For the comet assay, 50 μL of blood were transferred to a tube containing 1 mL of freshly prepared cold mincing buffer (Mg^+2^, Ca^+2^, and phenol free Hank's Balanced Salt Solution [Life Technologies, Carlsbad, CA] with 20 mM ethylenediamine tetraacetic acid (EDTA) pH 10.0 and 10% vol/vol fresh dimethyl sulfoxide [DMSO]) pH 7.47. The liver and the hippocampus, cerebellum, and frontal cortex sections of the brain were removed, rinsed with cold mincing buffer, and held on ice (≤5 min) until processed. Small portions (3–4 mm^3^) of each tissue were placed in tubes containing cold mincing solution and rapidly minced until finely dispersed. Blood and minced tissue samples were immediately flash frozen in liquid nitrogen and transferred to a −80°C freezer for a minimum of 1 week until shipment by overnight air courier on dry ice to the analytical laboratory (ILS, Research Triangle Park, NC).

For the MN assay, blood samples (~200 μL) were placed into EDTA tubes and immediately refrigerated. The samples were sent on the day of collection to ILS on cold packs via overnight air courier. Upon arrival, samples were diluted in anticoagulant (heparin) and fixed in ice‐cold methanol (Sigma‐Aldrich, St. Louis, MO) according to instructions provided with the MicroFlow^PLUS^ Kit (Litron Laboratories, Rochester, NY). Fixed samples were stored in a −80°C freezer for at least 3 days prior to analysis by flow cytometry.

### Comet Assay

Slides were prepared and analyzed as described previously (Hobbs et al. 2012; Recio et al. 2012) with some modifications. In a laboratory with controlled humidity (≤60%), samples were thawed on ice and a portion of the cell suspension was diluted with 0.5% low melting point agarose (Lonza, Walkersville, MD) dissolved in Dulbecco's phosphate buffer (Ca^+2^, Mg^+2^, and phenol free) at 37°C and layered onto each well of a 2‐well CometSlide™ (Trevigen, Gaithersburg, MD). Slides were prepared one tissue at a time, such that 35 slides were prepared at a time in 3 batches of 10 and 1 batch of 5, and each batch was immediately refrigerated to solidify the agarose and prevent deterioration of the samples. Once all slides per tissue had been prepared and refrigerated for at least 20 min (typically ≤2 hr for completion of an entire set), the slides were immersed in cold lysing solution (2.5 M NaCl, 100 mM Na_2_EDTA, 10 mM tris[hydroxymethyl]aminomethane, pH 10, containing freshly added 10% DMSO, and 1% Triton X‐100) overnight with refrigeration. After rinsing in 0.4 M Trizma base (pH 7.5), slides were treated with cold alkali solution (300 mM NaOH, 1 mM Na_2_EDTA, pH > 13) for 20 min to allow DNA unwinding, electrophoresed at 4–9°C for 20 min at 25 V (0.7 V/cm), with a current of approximately 300 mA, neutralized with Trizma base, dehydrated in absolute ethanol (Pharmco‐AAPER, Shelbyville, KY), and air‐dried. Slides from the same species, sex, and tissue were run together during electrophoresis and were placed randomly into the electrophoresis tank by exposure level and modulation to control for any possible variations in electrical field. Slides were stored at room temperature in a desiccator (relative humidity ≤60%) until stained and scored. NaCl, Na_2_EDTA, Triton X‐100, DMSO, and Trizma base were purchased from Sigma‐Aldrich; NaOH was purchased from Fisher Scientific (Pittsburgh, PA).

After staining with SYBR® Gold (Molecular Probes, Life Technologies, Grand Island, NY), slides, independently coded to mask treatment, were scored using Comet Assay IV Imaging Software, Version 4.3.1 (Perceptive Instruments, Suffolk, UK). DNA migration was quantified as % tail DNA (OECD 2016). Comets were classified as scorable, nonscorable, or “hedgehog.” Comets were classified as hedgehogs if they had no easily defined head, that is, all DNA appeared to be in the tail, or the head and tail appeared separated. Initially, % tail DNA was determined for 100 scorable comet figures per animal/tissue, standard practice at the time the study was conducted (prior to OECD Guideline 489). In addition, the frequency of hedgehogs was determined by tabulating the number of hedgehogs per 100 cells per animal/tissue, but hedgehog frequencies were not analyzed for statistical significance, in accordance with OECD Guideline 489. Although it has been proposed that hedgehogs are apoptotic cells, some studies strongly suggest that hedgehogs represent cells with high levels of repairable DNA damage (Rundell et al. 2003; Lorenzo et al. 2013), and it remains uncertain in the field as to what hedgehogs represent.

In the initial scoring of the rat samples, we noted that the range of % tail DNA values appeared truncated at ~ 65%. To better understand this observation, we reanalyzed the rat slides, scoring 150 cells/tissue/animal, as recommended by the OECD guideline (OECD 2016). In this second scoring exercise, we included analysis of scorable comet images that, upon visual inspection, appeared to be hedgehogs to determine if this affected the capture of DNA damage levels between 65 and 100% tail DNA. For the 150‐cell scoring method, because the % hedgehogs were not independently determined, the value was estimated by dividing the number of comets with ≥90% tail DNA by 150. Several mouse tissues were also reevaluated using the 150‐cell method for comparison. Although there was no concurrent positive control group (as is standard for all NTP chronic and subchronic animal toxicity tests), slides made with human lymphoblastoid TK6 cells treated with ethyl methanesulfonate were processed in parallel with each tissue set as an internal technical control for slide preparation, staining, and electrophoresis.

### Micronucleus Assay

Flow cytometric analysis of red blood cells was performed using MicroFlow^PLUS^ Kit reagents and a FACSCalibur™ dual‐laser bench top system (Becton Dickinson Biosciences, San Jose, CA) as described previously (Witt et al. 2008) and was consistent with OECD Test Guideline 474 (OECD 2014). Briefly, both immature erythrocytes (reticulocytes, RET) and mature erythrocytes were analyzed for the presence of MN. For each sample, 20,000 (±2,000) RET were analyzed and ~1 × 10^6^ mature erythrocytes were enumerated concurrently during micronucleated–RET (MN‐RET) analysis, allowing for calculation of the percentage of RET (%RET) among total erythrocytes as a measure of bone marrow toxicity.

### Data analysis

Data from both the comet and the MN assays, presented as mean ± standard error (SE), were analyzed using the same statistical methods (Kissling et al. 2007). Mean % tail DNA was calculated for each tissue per animal; likewise, mean MN–RET and MN–erythrocytes per 1,000 cells, as well as %RET, were calculated for each animal. Levene's test was used to determine if variances among treatment groups were equal at significance level 0.05. When variances were equal, linear regression analysis was used to test for trend and Williams' test was used to evaluate pairwise differences between each treated group and the control. When variances were unequal, Jonckheere's test was used to evaluate linear trend and Dunn's test was used to assess the significance of pairwise differences of each treated group with the control group. To maintain the overall significance level at 0.05, the trend as well as the pairwise differences were declared statistically significant if *P* < 0.025. A result was considered positive if the trend test was significant and at least one dose group was significantly elevated over the control, or if two or more dose groups were significantly increased over the corresponding control. A response was considered equivocal if only the trend test was significant or only a single dose group was significantly increased over the control. In the absence of either a significant trend or a significantly elevated dose group, the result was considered negative.

## RESULTS

### Comet Assay

**Figure 1:**
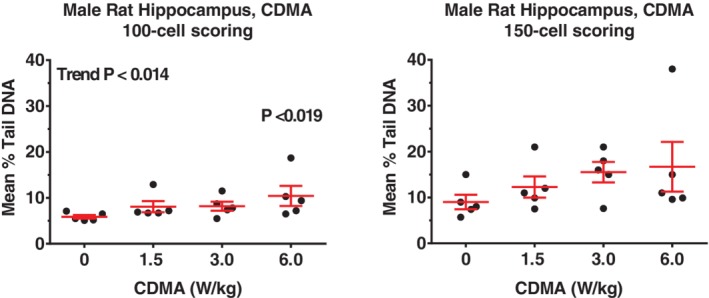
(A,B) Male rat hippocampus, CDMA, was the only rat tissue judged to be positive in the comet assay when using the 100‐cell scoring approach (A). Central horizontal bar indicates mean % tail DNA; upper and lower error bars indicate SE.

**Figure 2:**
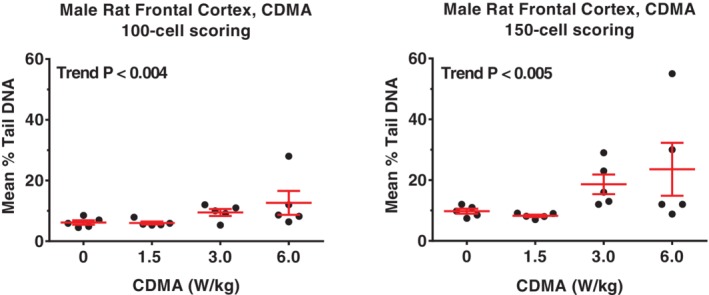
(A,B) Male rat frontal cortex, CDMA, was judged to be equivocal in the comet assay using the 100‐cell scoring approach (A); a similar result was obtained using the 150‐cell scoring approach (B). Central horizontal bar indicates mean % tail DNA; upper and lower error bars indicate SE.

**Figure 3:**
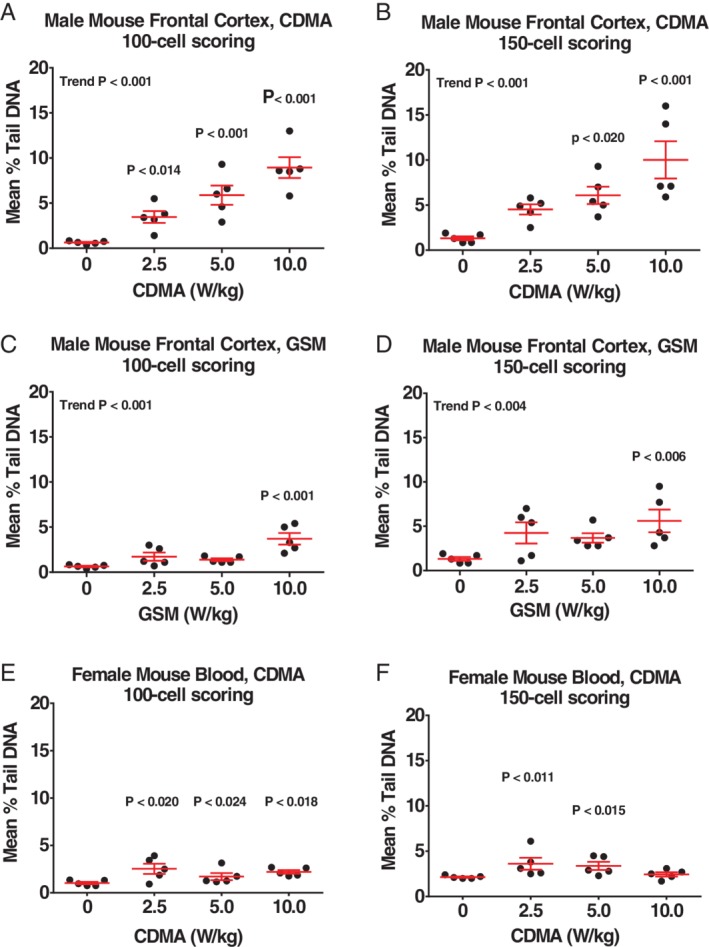
(A–F) Mouse tissues judged to be positive in the comet assay using the 100‐cell scoring approach. Central horizontal bar indicates mean % tail DNA; upper and lower error bars indicate SE.

Eight hundred tissue samples were analyzed for % tail DNA in the comet assay. The mean % tail DNA, SE, and statistical outcomes for pairwise and trend comparisons are shown for all 40 sets of tissues (5 tissues × 8 conditions of the study) in Tables 1, 2, 3, 4, 5, 6, 7, 8. Results are reported based on the standard 100‐cell scoring approach in use at the time that the data were collected. Data obtained using the 150‐cell scoring approach (OECD 2016) are noted for the few instances where results differed between the two methods. In addition, results that were either positive or equivocal are presented in figures to illustrate interanimal variability in response, and to compare the 100‐ versus 150‐cell scoring results (Figs. 1, 2, 3). Samples were not removed from analysis unless a technical issue was identified with acquisition of the sample, or if the result was considered to be biologically implausible, as apparent outliers or influential data points could represent true biological variability. Of the 800 tissue samples that were analyzed for % tail DNA, three samples were omitted from analysis. Two samples, female rat hippocampal tissue exposed to 1.5 W/kg GSM and female rat hippocampal tissue exposed to 3.0 W/kg, were omitted due to a labeling error that occurred during necropsy. A sample of hippocampal tissue from a sham‐exposed female rat was omitted because it had a biologically implausible value of 56.1% tail DNA.

**Table 1:** DNA damage in Male Sprague Dawley Rats Exposed to CDMA‐Modulated Cell Phone Radiofrequency Radiation (900 MHz) for 19 Weeks^a^

	Dose (W/kg)	% Tail DNA (100 cells)^b^	*P* value^c^	% Hedgehogs (100 cells)^b^	% Tail DNA (150 cells)^b^	*P* value	% Hedgehogs (150 cells)^b^^,^ d
Frontal cortex							
	0^e^	6.18 ± 0.72		2.00 ± 0.71	9.73 ± 0.81		0.27 ± 0.27
CDMA	1.5	6.00 ± 0.48	1.000	1.00 ± 0.77	8.24 ± 0.39	1.000	0.13 ± 0.13
	3.0	9.51 ± 1.17	0.081	10.60 ± 3.89	18.77 ± 3.27	0.043	2.53 ± 1.29
	6.0	12.78 ± 3.96	0.049	12.20 ± 6.84	23.62 ± 8.66	0.092	3.20 ± 1.72
		*P* = 0.004			*P* = 0.005^f^		
Hippocampus							
	0	5.88 ± 0.39		3.40 ± 1.21	8.99 ± 1.55		1.07 ± 0.45
CDMA	1.5	8.06 ± 1.20	0.135	3.80 ± 2.33	12.27 ± 2.21	0.244	0.40 ± 0.27
	3.0	8.16 ± 0.98	0.151	6.20 ± 2.56	15.46 ± 2.25	0.107	2.53 ± 0.90
	6.0	10.42 ± 2.18	0.019	4.40 ± 2.98	16.77 ± 5.44	0.069	2.40 ± 1.44
		*P* = 0.014			*P* = 0.043		
Cerebellum							
	0	5.57 ± 0.92		0.40 ± 0.24	4.90 ± 0.82		0 ± 0
CDMA	1.5	5.60 ± 0.71	1.000	1.80 ± 0.80	6.33 ± 1.00	0.681	0.27 ± 0.16
	3.0	10.70 ± 3.66	0.504	9.40 ± 6.81	13.75 ± 6.01	0.504	2.93 ± 2.20
	6.0	10.58 ± 3.52	0.731	8.00 ± 3.91	15.86 ± 5.91	0.163	2.40 ± 1.07
		*P* = 0.156			*P* = 0.061		
Liver							
	0	13.81 ± 2.88		33.60 ± 17.89	25.71 ± 8.71		1.73 ± 1.73
CDMA	1.5	22.99 ± 2.77	0.081	68.60 ± 15.70	55.41 ± 7.91	0.136	14.67 ± 5.57
	3.0	16.04 ± 2.14	0.098	7.80 ± 0.86	19.11 ± 2.28	0.164	0.80 ± 0.49
	6.0	20.79 ± 3.10	0.057	41.10 ± 14.80	40.01 ± 7.90	0.114	9.07 ± 7.10
		*P* = 0.154			*P* = 0.385		
Peripheral blood leukocytes							
	0	1.48 ± 0.29		0.20 ± 0.20	0.69 ± 0.20		0 ± 0
CDMA	1.5	1.22 ± 0.45	0.596	0.80 ± 0.80	1.16 ± 0.47	0.295	0 ± 0
	3.0	2.13 ± 0.34	0.156	0.40 ± 0.40	1.83 ± 0.74	0.121	0.13 ± 0.13
	6.0	2.08 ± 0.43	0.166	1.40 ± 1.17	2.57 ± 0.80	0.026	0 ± 0
		*P* = 0.071			*P* = 0.012		

a Exposure began *in utero* on GD5.

b Mean ± SE.

c Pairwise comparison with the sham control group; exposed group values are significant at *P* ≤ 0.025 by Williams' or Dunn's test.

d A comet figure was considered a hedgehog if ≥90% DNA was in the tail. % Hedgehogs = number of comets with ≥90% tail DNA/150.

e Sham control; no exposure to CDMA‐modulated cell phone RFR.

f Dose‐related trend derived from one‐tailed linear regression or Jonckheere's test; the trend is significant when *P* ≤ 0.025.

**Table 2:** DNA Damage in Male Sprague Dawley Rats Exposed to GSM‐Modulated Cell Phone Radiofrequency Radiation (900 MHz) for 19 Weeks^a^

	Dose (W/kg)	% Tail DNA (100 cells)^b^	*P* value	% Hedgehogs (100 cells)^b^	% Tail DNA (150 cells)^b^	*P* value^c^	% Hedgehogs (150 cells)^b^^,^ d
Frontal cortex							
	0^e^	6.18 ± 0.72		2.00 ± 0.71	9.73 ± 0.81		0.27 ± 0.27
GSM	1.5	6.98 ± 0.42	0.465	1.40 ± 0.51	11.96 ± 1.65	0.634	0.40 ± 0.27
	3.0	8.66 ± 1.96	0.247	8.20 ± 2.69	17.98 ± 5.12	0.545	1.20 ± 0.57
	6.0	6.30 ± 0.32	1.000	3.00 ± 1.55	9.57 ± 1.57	1.000	1.30 ± 0.13
		*P* = 0.343			*P* = 0.500^f^		
Hippocampus							
	0	5.88 ± 0.39		3.40 ± 1.21	8.99 ± 1.55		1.07 ± 0.45
GSM	1.5	11.82 ± 2.68	0.092	4.80 ± 2.84	17.24 ± 4.09	0.186	0.27 ± 0.16
	3.0	9.64 ± 1.27	0.111	4.80 ± 1.53	14.77 ± 2.54	0.227	1.47 ± 0.57
	6.0	11.69 ± 3.92	0.072	10.20 ± 7.98	21.32 ± 9.55	0.080	3.60 ± 2.03
		*P* = 0.103			*P* = 0.076		
Cerebellum							
	0	5.57 ± 0.92		0.40 ± 0.24	4.90 ± 0.82		0 ± 0
GSM	1.5	7.36 ± 2.48	0.295	2.40 ± 1.91	9.43 ± 4.69	0.190	1.33 ± 1.17
	3.0	6.37 ± 0.77	0.354	3.40 ± 1.17	8.66 ± 2.17	0.232	1.47 ± 0.68
	6.0	8.48 ± 1.85	0.149	5.00 ± 2.86	12.11 ± 3.89	0.088	1.07 ± 1.07
		*P* = 0.132			*P* = 0.076		
Liver							
	0	13.81 ± 2.88		33.60 ± 17.89	25.71 ± 8.71		1.73 ± 1.73
GSM	1.5	13.26 ± 2.38	0.547	21.00 ± 12.30	23.27 ± 9.43	0.539	4.13 ± 3.64
	3.0	13.09 ± 2.32	0.634	28.40 ± 15.07	25.15 ± 8.43	0.604	0.40 ± 0.40
	6.0	14.49 ± 2.71	0.536	24.80 ± 16.13	28.25 ± 10.55	0.534	4.93 ± 3.94
		*P* = 0.404			*P* = 0.390		
Peripheral blood leukocytes							
	0	1.48 ± 0.29		0.20 ± 0.20	0.69 ± 0.20		0 ± 0
GSM	1.5	1.83 ± 0.63	0.352	3.20 ± 2.71	3.97 ± 2.75	0.146	0.27 ± 0.27
	3.0	1.78 ± 0.33	0.419	1.20 ± 0.49	1.97 ± 0.35	0.021	0 ± 0
	6.0	1.50 ± 0.27	0.446	0.40 ± 0.24	1.28 ± 0.23	0.272	0 ± 0
		*P* = 0.550			*P* = 0.089		

a Exposure began *in utero* on GD5.

b Mean ± SE.

c Pairwise comparison with the sham control group; exposed group values are significant at *P* ≤ 0.025 by Williams' or Dunn's test.

d A comet figure was considered a hedgehog if ≥90% DNA was in the tail. % Hedgehogs = number of comets with ≥90% tail DNA/150.

e Sham control; no exposure to GSM‐modulated cell phone RFR.

f Dose‐related trend derived from one‐tailed linear regression or Jonckheere's test; the trend is significant when *P* ≤ 0.025.

**Table 3:** DNA Damage in Female Sprague Dawley Rats Exposed to CDMA‐Modulated Cell Phone Radiofrequency Radiation (900 MHz) for 19 Weeks^a^

	Dose (W/kg)	% Tail DNA (100 cells)^b^	*P* value	% Hedgehogs (100 cells)^b^	% Tail DNA (150 cells)^b^	*P* value^c^	% Hedgehogs (150 cells)^b^^,^ d
Frontal cortex							
	0^e^	7.03 ± 1.21		3.80 ± 1.46	12.23 ± 2.18		0.40 ± 0.16
CDMA	1.5	12.70 ± 5.15	0.205	19.00 ± 15.04	25.37 ± 12.96	0.782	8.67 ± 7.67
	3.0	9.50 ± 2.27	0.249	9.80 ± 5.12	18.70 ± 5.28	0.634	1.87 ± 0.88
	6.0	13.00 ± 3.63	0.150	25.40 ± 11.44	33.49 ± 11.14	0.092	7.20 ± 5.62
		*P* = 0.166^f^			*P* = 0.035		
Hippocampus							
	0^g^	13.14 ± 1.20		9.00 ± 2.58	18.08 ± 1.30		0.83 ± 0.32
CDMA	1.5	14.94 ± 0.70	0.346	8.40 ± 1.96	20.58 ± 2.06	0.531	1.07 ± 0.34
	3.0	15.24 ± 1.97	0.379	9.40 ± 2.89	20.63 ± 1.92	0.382	1.33 ± 0.21
	6.0	19.11 ± 5.27	0.126	21.20 ± 11.12	29.55 ± 9.44	0.218	6.53 ± 5.23
		*P* = 0.080			*P* = 0.068		
Cerebellum							
	0	5.94 ± 0.98		3.80 ± 1.07	4.93 ± 1.09		0 ± 0
CDMA	1.5	4.91 ± 0.58	0.671	2.00 ± 1.05	4.61 ± 1.61	0.621	0.53 ± 0.53
	3.0	5.46 ± 0.83	0.747	2.00 ± 0.63	3.89 ± 0.43	0.709	0.13 ± 0.13
	6.0	5.86 ± 0.84	0.650	1.20 ± 0.37	5.88 ± 0.63	0.342	0.27 ± 0.16
		*P* = 0.421			*P* = 0.249		
Liver							
	0	10.09 ± 0.87		7.00 ± 1.87	12.41 ± 1.64		0.13 ± 0.13
CDMA	1.5	15.26 ± 3.35	0.634	33.40 ± 15.11	26.15 ± 8.57	0.145	4.00 ± 3.67
	3.0	11.49 ± 2.05	1.000	12.40 ± 3.59	16.17 ± 2.17	0.176	0.67 ± 0.42
	6.0	18.35 ± 3.44	0.163	31.40 ± 12.33	26.65 ± 6.91	0.059	2.00 ± 1.17
		*P* = 0.113			*P* = 0.102		
Peripheral blood leukocytes							
	0	3.15 ± 0.40		0.20 ± 0.20	3.32 ± 0.09		0.13 ± 0.13
CDMA	1.5	3.77 ± 1.19	0.371	1.20 ± 0.80	4.45 ± 1.53	1.000	0.40 ± 0.27
	3.0	4.13 ± 0.54	0.361	0.40 ± 0.40	3.94 ± 0.40	0.465	0.13 ± 0.13
	6.0	6.06 ± 2.18	0.082	9.80 ± 8.81	12.76 ± 7.59	0.028	2.93 ± 2.77
		*P* = 0.048			*P* = 0.013		

a Exposure began *in utero* on GD5.

b Mean ± SE.

c Pairwise comparison with the sham control group; exposed group values are significant at *P* ≤ 0.025 by Williams' or Dunn's test.

d A comet figure was considered a hedgehog if ≥90% DNA was in the tail. % Hedgehogs = number of comets with ≥90% tail DNA/150.

e Sham control; no exposure to CDMA‐modulated cell phone RFR.

f Dose‐related trend derived from one‐tailed linear regression or Jonckheere's test; the trend is significant when *P* ≤ 0.025.

g *n* = 4.

**Table 4:** DNA Damage in Female Sprague Dawley Rats Exposed to GSM‐Modulated Cell Phone Radiofrequency Radiation (900 MHz) for 19 Weeks^a^

	Dose (W/kg)	% Tail DNA (100 cells)^b^	*P* value	% Hedgehogs (100 cells)^b^	% Tail DNA (150 cells)^b^	*P* value^c^	% Hedgehogs (150 cells)^b^^,^ d
Frontal cortex							
	0^e^	7.03 ± 1.21		3.80 ± 1.46	12.23 ± 2.18		0.40 ± 0.16
GSM	1.5	4.87 ± 0.47	0.820	2.20 ± 0.73	6.28 ± 1.00	0.856	0 ± 0
	3.0	6.18 ± 0.67	0.843	5.60 ± 2.36	9.83 ± 1.11	0.877	0.67 ± 0.21
	6.0	6.74 ± 0.74	0.723	6.40 ± 2.73	13.74 ± 2.79	0.376	0.13 ± 0.13
		*P* = 0.386			*P* = 0.137^f^		
Hippocampus							
	0^g^	13.14 ± 1.20		9.00 ± 2.58	18.08 ± 1.30		0.83 ± 0.32
GSM	1.5^g^	13.22 ± 1.56	0.936	7.25 ± 3.20	17.54 ± 3.59	1.000	1.50 ± 1.29
	3.0^g^	17.67 ± 3.64	0.351	19.50 ± 7.89	28.08 ± 7.00	0.662	3.66 ± 1.40
	6.0	13.21 ± 1.03	1.000	10.00 ± 3.81	18.19 ± 3.35	1.000	2.93 ± 1.53
		*P* = 0.334			*P* = 0.534		
Cerebellum							
	0	5.94 ± 0.98		3.80 ± 1.07	4.93 ± 1.09		0 ± 0
GSM	1.5	5.69 ± 0.75	0.662	2.00 ± 0.71	5.11 ± 0.63	0.731	0 ± 0
	3.0	4.62 ± 0.85	0.749	0.60 ± 0.24	3.51 ± 0.74	1.000	0 ± 0
	6.0	6.62 ± 0.96	0.381	2.40 ± 1.03	6.54 ± 2.33	1.000	0.27 ± 0.16
		*P* = 0.302			*P* = 0.705		
Liver							
	0	10.09 ± 0.87		7.00 ± 1.87	12.41 ± 1.64		0.13 ± 0.13
GSM	1.5	9.91 ± 2.60	1.000	13.20 ± 11.23	17.05 ± 7.24	1.000	0.93 ± 0.62
	3.0	9.46 ± 2.07	1.000	17.00 ± 14.76	14.06 ± 5.68	1.000	0.27 ± 0.16
	6.0	18.99 ± 6.20	1.000	35.20 ± 19.42	26.03 ± 10.69	1.000	4.00 ± 3.23
		*P* = 0.394			*P* = 0.580		
Peripheral blood leukocytes							
	0	3.15 ± 0.40		0.20 ± 0.20	3.32 ± 0.09		0.13 ± 0.13
GSM	1.5	2.80 ± 0.33	0.593	0.80 ± 0.49	3.07 ± 0.43	1.000	0.27 ± 0.16
	3.0	3.39 ± 0.68	0.447	0.60 ± 0.24	2.82 ± 0.52	1.000	0.13 ± 0.13
	6.0	3.93 ± 0.63	0.203	1.00 ± 0.32	3.86 ± 0.76	1.000	0.40 ± 0.16
		*P* = 0.093			*P* = 0.580		

a Exposure began in utero on GD5.

b Mean ± SE.

c Pairwise comparison with the sham control group; exposed group values are significant at *P* ≤ 0.025 by Williams' or Dunn's test.

d A comet figure was considered a hedgehog if ≥90% DNA was in the tail. % Hedgehogs = number of comets with ≥90% tail DNA/150.

e Sham control; no exposure to GSM‐modulated cell phone RFR.

f Dose‐related trend derived from one‐tailed linear regression or Jonckheere's test; the trend is significant when *P* ≤ 0.025.

g *n* = 4.

**Table 5:** DNA Damage in Male B6C3F1/N Mice Exposed to CDMA‐Modulated Cell Phone Radiofrequency Radiation (1,900 MHz) for 14 Weeks^a^

	Dose (W/kg)	% Tail DNA (100 cells)^b^	*P* value^c^	% Hedgehogs (100 cells)^b^	% Tail DNA (150 cells)^b^	*P* value	% Hedgehogs (150 cell)^d^
Frontal cortex							
	0^e^	0.63 ± 0.08		0.40 ± 0.24	1.32 ± 0.21		0 ± 0
CDMA	2.5	3.46 ± 0.65	0.014	0.60 ± 0.40	4.52 ± 0.57	0.131	0 ± 0
	5.0	5.88 ± 1.06	0.001	0.60 ± 0.24	6.06 ± 0.96	0.018	0 ± 0
	10.0	8.85 ± 1.09	0.001	4.40 ± 1.69	10.04 ± 2.08	0.001	0.53 ± 0.39
		*P* = 0.001^f^			*P* = 0.001		
Hippocampus							
	0	7.69 ± 2.00		1.20 ± 0.58			
CDMA	2.5	9.59 ± 4.33	0.521	5.40 ± 2.11			
	5.0	6.44 ± 1.21	0.606	2.80 ± 0.97			
	10.0	6.38 ± 0.93	0.641	4.40 ± 2.27			
		*P* = 0.740					
Cerebellum							
	0	5.48 ± 1.30		1.80 ± 0.80			
CDMA	2.5	7.35 ± 2.47	0.339	4.40 ± 2.06			
	5.0	7.87 ± 2.80	0.404	4.60 ± 2.34			
	10.0	5.43 ± 2.43	0.431	1.60 ± 0.93			
		*P* = 0.554					
Liver							
	0	16.30 ± 2.21		6.80 ± 2.82			
CDMA	2.5	20.27 ± 5.53	1.000	21.60 ± 16.88			
	5.0	16.15 ± 1.15	1.000	11.00 ± 3.77			
	10.0	16.43 ± 0.83	1.000	7.20 ± 1.11			
		*P* = 0.368					
Peripheral blood leukocytes		1.60 ± 0.68		0.40 ± 0.24			
	0						
		2.10 ± 0.50	0.449	1.20 ± 0.58			
CDMA	2.5	1.30 ± 0.28	0.527	0.40 ± 0.24			
	5.0	2.86 ± 0.26	0.046	1.40 ± 0.87			
	10.0						
		*P* = 0.057					

a Exposure began at ~5 weeks of age.

b Mean ± SE.

c Pairwise comparison with the sham control group; exposed group values are significant at *P* ≤ 0.025 by Williams' or Dunn's test.

d A comet figure was considered a hedgehog if ≥90% DNA was in the tail. % Hedgehogs = number of comets with ≥90% tail DNA/150.

e Sham control; no exposure to CDMA‐modulated cell phone RFR.

f Dose‐related trend derived from one‐tailed linear regression or Jonckheere's test; the trend is significant when *P* ≤ 0.025.

**Table 6:** DNA Damage in Male B6C3F1/N Mice Exposed to GSM‐Modulated Cell Phone Radiofrequency Radiation (1,900 MHz) for 14 Weeks^a^

	Dose (W/kg)	% Tail DNA (100 cells)^b^	*P* value^c^	% Hedgehogs (100 cells)^b^	% Tail DNA (150 cells)^b^	*P* value	% Hedgehogs (150 cell)^d^
Frontal cortex							
	0^e^	0.63 ± 0.08		0.40 ± 0.24	1.32 ± 0.21		0 ± 0
GSM	2.5	1.71 ± 0.46	0.081	1.80 ± 0.97	4.25 ± 1.20	0.063	0.13 ± 0.13
	5.0	1.39 ± 0.15	0.081	1.60 ± 0.81	3.69 ± 0.53	0.063	0 ± 0
	10.0	3.73 ± 0.65	0.001	1.00 ± 0.45	5.60 ± 1.28	0.006	0.13 ± 0.13
		*P* = 0.001^f^			*P* = 0.004		
Hippocampus							
	0	7.69 ± 2.00		1.20 ± 0.58			
GSM	2.5	8.74 ± 1.93	0.514	5.40 ± 2.11			
	5.0	7.17 ± 1.08	0.598	2.20 ± 0.97			
	10.0	6.90 ± 1.19	0.633	5.40 ± 2.54			
		*P* = 0.720					
Cerebellum							
	0	5.48 ± 1.30		1.80 ± 0.80			
GSM	2.5	3.66 ± 0.30	0.831	3.00 ± 1.38			
	5.0	3.90 ± 0.59	0.896	1.80 ± 0.92			
	10.0	3.85 ± 1.08	0.919	3.40 ± 1.50			
		*P* = 0.838					
Liver							
	0	16.30 ± 2.21		6.80 ± 2.82			
GSM	2.5	17.66 ± 1.89	0.469	8.20 ± 3.84			
	5.0	15.40 ± 1.20	0.549	6.60 ± 1.96			
	10.0	18.94 ± 2.00	0.213	12.80 ± 4.40			
		*P* = 0.198					
Peripheral blood leukocytes							
	0	1.60 ± 0.68		0.40 ± 0.24			
GSM	2.5	1.85 ± 0.96	0.416	1.20 ± 1.20			
	5.0	1.75 ± 0.37	0.491	1.00 ± 0.55			
	10.0	1.85 ± 0.24	0.494	0.80 ± 0.58			
		*P* = 0.408					

a Exposure began at ~5 weeks of age.

b Mean ± SE.

c Pairwise comparison with the sham control group; exposed group values are significant at *P* ≤ 0.025 by Williams' or Dunn's test.

d A comet figure was considered a hedgehog if ≥90% DNA was in the tail. % Hedgehogs = number of comets with ≥90% tail DNA/150.

e Sham control; no exposure to GSM‐modulated cell phone RFR.

f Dose‐related trend derived from one‐tailed linear regression or Jonckheere's test; the trend is significant when *P* ≤ 0.025.

**Table 7:** DNA Damage in Female B6C3F1/N Mice Exposed to CDMA‐Modulated Cell Phone Radiofrequency Radiation (1,900 MHz) for 14 Weeks^a^

	Dose (W/kg)	% Tail DNA (100 cells)^b^	*P* value^c^	% Hedgehogs (100 cells)^b^	% Tail DNA (150 cells)^b^	*P* value	% Hedgehogs (150 cell)^d^
Frontal cortex							
	0^e^	8.11 ± 2.13		3.40 ± 1.47			
CDMA	2.5	4.88 ± 0.55	0.911	0.80 ± 0.49			
	5.0	4.89 ± 0.57	0.955	1.20 ± 0.49			
	10.0	4.80 ± 0.90	0.968	0.80 ± 0.58			
		*P* = 0.935^f^					
Hippocampus							
	0	8.15 ± 1.65		2.60 ± 1.69			
CDMA	2.5	5.76 ± 1.00	0.839	1.80 ± 0.80			
	5.0	5.22 ± 1.02	0.903	1.20 ± 0.58			
	10.0	5.34 ± 1.82	0.925	2.20 ± 0.97			
		*P* = 0.892					
Cerebellum							
	0	5.88 ± 0.85		0.20 ± 0.20			
CDMA	2.5	6.78 ± 1.67	0.296	1.75 ± 1.03			
	5.0	8.39 ± 1.13	0.194	0.20 ± 0.20			
	10.0	6.73 ± 0.77	0.207	0.40 ± 0.40			
		*P* = 0.298					
Liver							
	0	5.48 ± 0.60		0.60 ± 0.40	4.34 ± 0.60		0 ± 0
CDMA	2.5	7.54 ± 0.90	0.034	1.00 ± 0.45	6.20 ± 0.99	0.050	0 ± 0
	5.0	7.36 ± 0.72	0.041	4.40 ± 2.11	8.30 ± 0.92	0.009	0 ± 0
	10.0	7.63 ± 0.59	0.030	2.00 ± 0.77	6.14 ± 0.26	0.009	0 ± 0
		*P* = 0.050			*P* = 0.100		
Peripheral blood leukocytes							
	0	1.03 ± 0.13		0.20 ± 0.20	2.15 ± 0.08		0 ± 0
CDMA	2.5	2.52 ± 0.54	0.020	2.00 ± 1.14	3.62 ± 0.66	0.011	0 ± 0
	5.0	1.71 ± 0.37	0.024	0 ± 0	3.39 ± 0.45	0.015	0.13 ± 0.13
	10.0	2.20 ± 0.19	0.018	0.20 ± 0.20	2.45 ± 0.24	0.428	0 ± 0
		*P* = 0.085			*P* = 0.173		

a Exposure began at ~5 weeks of age.

b Mean ± SE.

c Pairwise comparison with the sham control group; exposed group values are significant at *P* ≤ 0.025 by Williams' or Dunn's test.

d A comet figure was considered a hedgehog if ≥90% DNA was in the tail. % Hedgehogs = number of comets with ≥90% tail DNA/150.

e Sham control; no exposure to CDMA‐modulated cell phone RFR.

f Dose‐related trend derived from one‐tailed linear regression or Jonckheere's test; the trend is significant when *P* ≤ 0.025.

**Table 8:** DNA Damage in Female B6C3F1/N Mice Following Exposure to GSM‐Modulated Cell Phone Radiofrequency Radiation (1,900 MHz) for 14 Weeks^a^

	Dose (W/kg)	% Tail DNA (100 cells)^b^	*P* value^c^	% Hedgehogs (100 cells)^b^	% Tail DNA (150 cells)^b^	*P* value	% Hedgehogs (150 cell)^d^
Frontal cortex							
	0^e^	8.11 ± 2.13		3.40 ± 1.47			
GSM	2.5	7.33 ± 0.90	0.657	1.00 ± 0.45			
	5.0	7.69 ± 1.98	0.744	2.00 ± 0.84			
	10.0	5.74 ± 0.62	0.779	1.00 ± 0.32			
		*P* = 0.861^f^					
Hippocampus							
	0	8.15 ± 1.65		2.60 ± 1.69			
GSM	2.5	6.23 ± 1.00	0.866	0.80 ± 0.58			
	5.0	4.54 ± 1.29	0.923	1.20 ± 0.58			
	10.0	5.22 ± 1.23	0.942	1.60 ± 1.36			
		*P* = 0.933					
Cerebellum							
	0	5.88 ± 0.85		0.20 ± 0.20			
GSM	2.5	6.56 ± 1.22	1.000	1.20 ± 0.73			
	5.0	5.26 ± 0.59	1.000	0.60 ± 0.40			
	10.0	6.54 ± 1.71	1.000	1.80 ± 0.73			
		*P* = 0.606					
Liver							
	0	5.48 ± 0.60		0.60 ± 0.40	4.34 ± 0.60		0 ± 0
GSM	2.5	7.06 ± 0.61	0.096	3.40 ± 1.17	7.44 ± 0.48	0.027	0 ± 0
	5.0	6.36 ± 0.25	0.117	1.20 ± 0.37	5.45 ± 0.96	0.032	0 ± 0
	10.0	6.47 ± 0.79	0.124	2.60 ± 1.33	6.52 ± 0.75	0.030	0 ± 0
		*P* = 0.249			*P* = 0.133		
Peripheral blood leukocytes							
	0	1.03 ± 0.13		0.20 ± 0.20	2.15 ± 0.08		0 ± 0
GSM	2.5	1.25 ± 0.44	0.335	0.20 ± 0.20	2.58 ± 0.35	0.504	0 ± 0
	5.0	1.17 ± 0.08	0.400	0 ± 0	2.23 ± 0.19	1.000	0 ± 0
	10.0	1.32 ± 0.34	0.316	0 ± 0	2.28 ± 0.51	1.000	0 ± 0
		*P* = 0.266			*P* = 0.657		

a Exposure began at ~5 weeks of age.

b Mean ± SE.

c Pairwise comparison with the sham control group; exposed group values are significant at *P* ≤ 0.025 by Williams' or Dunn's test.

d A comet figure was considered a hedgehog if ≥90% DNA was in the tail. % Hedgehogs = number of comets with ≥90% tail DNA/150.

e Sham control; no exposure to GSM‐modulated cell phone RFR.

f Dose‐related trend derived from one‐tailed linear regression or Jonckheere's test; the trend is significant when *P* ≤ 0.025.

In rats, the only clear positive result was observed in hippocampus cells of male rats exposed to the CDMA modulation when evaluated using the 100‐cell scoring approach (Table 1; Fig. 1A,B). Although the levels of DNA damage in hippocampus cells were also increased in an exposure‐related fashion using the 150‐cell scoring approach, the increases did not meet our criteria for statistical significance (Table 1). Equivocal results were obtained for the frontal cortex (CDMA) of male rats using both scoring approaches (Table 1; Fig. 2A, B). For male rat blood leukocytes (both modulations), results from scoring 100 cells were negative; however, equivocal responses were seen with the 150‐cell method based on a significant trend test (*P* = 0.012) or pairwise test (*P* = 0.021) for CDMA‐ and GSM‐exposed rats, respectively (Tables 1 and 2). No statistically significant increases in % tail DNA were observed in any of the samples from female rats exposed to either modulation (Tables 3 and 4). Although it would appear that an equivocal result was obtained for CMDA‐exposed female rat blood leukocytes using the 150‐cell scoring approach (Table 3), this result was driven by a single animal in the high exposure (6 W/kg) group.

In mice, positive results were obtained with both scoring approaches in frontal cortex of male mice (CDMA and GSM) (Tables 5 and 6; Fig. 3A–D) and blood leukocytes of female mice (CDMA) (Table 7; Fig. 3E,F). Scoring 150 cells resulted in a positive response in liver of female mice exposed to CDMA; a similar pattern of response was seen with the 100‐cell scoring method, but none of the increases met our criteria for significance (Table 7). No statistically significant increases in % tail DNA were observed in any of the samples from female mice exposed to the GSM modulation (Table 8).

In general, for those data sets that were scored using both methods (100‐ and 150‐cell scoring methods), similar conclusions were reached when considering positive or equivocal results (see Supporting Information Fig. S1A–D for examples) except for hippocampus from male rats (CDMA) (Table 1), blood leukocytes from male rats (CDMA and GSM) (Tables 1 and 2), and liver from female mice (CDMA) (Table 7). In summary, 8 of 40 tissue sets exhibited positive or equivocal results when assessed using the 100‐ or 150‐cell scoring approaches.

In all instances, where both methods were used, the 150‐cell method that included all scorable cells, even those that visually appeared to be hedgehogs before software analysis, revealed a much broader spectrum of DNA damage than the 100‐cell method that excluded all apparent hedgehogs (Supporting Information Figs. S2A–D and S3A–D).

We noticed considerable interanimal variability in % tail DNA in both sexes of mice and rats. To rule out any influence from technical artifacts or protocol features, % tail DNA values for all tissues and % hedgehogs for the rat tissues were correlated to the position of slides in the electrophoresis chambers, the interval from exposure cessation to tissue collection, and the date of slide preparation. No patterns in the level of observed DNA damage emerged for any of these variables. To investigate the interanimal variability more closely, we plotted the % tail DNA response data for all tissues using the 100‐cell data set. The median % tail DNA was included in each plot as a measure of central tendency in the distribution (see Supporting Information Fig. S4A–D). We found that % tail DNA values were relatively small (<5%) in blood leukocytes in both sexes and species, while the other four tissues exhibited a much greater interanimal variability in response with % tail DNA values that exceeded 30% in some cases. Female mice generally displayed less variability in response than male mice in the hippocampus, cerebellum, and liver. Female rats exposed to RFR also seemed to show less variability in response than male rats exposed to RFR in the cerebellum.

### Micronucleus Assay

The MN assay data are reported in Supporting Information Tables S1 and S2. For male mice exposed to CDMA, although a significant trend was observed for MN–RET (*P* = 0.013), the absolute increase was quite small (the mean MN–RET for sham exposure was 2.55 vs. 2.93 for the 10 W/kg exposure) and within the laboratory's historical control range (1.66–3.06), and no corresponding increase was observed in the mature erythrocyte population that should be in steady‐state equilibrium after continuous subchronic exposure. Thus, the overall MN assay result for male mice exposed to CDMA was considered to be negative. No other significant effects were seen in rats or mice exposed to either modulation of RFR.

### RFR Exposure

The power levels for RFR exposure were adjusted based on the average weight of all animals in a chamber. Due to normal variations in animal weights, the actual SAR in individual animals differed slightly among animals in the same exposure chamber (Wyde et al. 2018). These minor deviations were considered to have negligible effect, as no correlations between actual individual animal SAR and comet assay outcomes were seen in any of several tissues, including brain, that were examined to evaluate possible associations (data not shown).

## DISCUSSION

The two main RFR modulations used for cellular telephone communication worldwide, CDMA and GSM, were tested by the NTP in the 2‐year rodent cancer bioassay. The reverberation chambers used to expose the animals for the bioassay were designed by physicists and engineers from NIST and IT'IS in collaboration with the NTP to overcome confounding factors that have limited the interpretation of other RFR studies. As a component of the bioassay, we examined the potential for RFR to induce DNA damage as measured by the comet assay and chromosomal damage as measured by the peripheral blood erythrocyte MN assay. Although results of the MN assays were negative, significant increases in the levels of DNA damage measured by the comet assay were seen in several tissues from rats and mice, indicating that RFR may be capable of causing increases in DNA damage.

DNA damage was primarily observed in brain tissue from male rats and mice exposed to RFR. Using the 100‐cell scoring approach, the hippocampus of CDMA‐exposed male rats showed a significant, exposure‐related increase in % tail DNA, while no tissues in exposed female rats were found to have significant increases in % tail DNA compared to controls. Male mice exhibited significant CDMA exposure‐related increases in % tail DNA compared to controls at all exposure levels in the frontal cortex, and a GSM exposure‐related increase in % tail DNA compared to controls at the highest exposure level in the frontal cortex. Female mice showed small, but statistically significant, increases in % tail DNA compared to controls at all exposure levels in blood. No other potentially exposure‐related patterns were apparent based on visual inspection of the % tail DNA data (see Figs. 1, 2, 3). A larger number of animals per treatment group may have improved the ability to detect increases in DNA damage; however, the size of the reverberation chambers limited the number of animals that could be used for genetic toxicity testing to 5 per treatment group, which is the standard for comet assay studies conducted at the NTP and consistent with OECD recommendations (Hartmann et al. 2003; OECD 2016).

A limitation in this study is the absence of histopathological assessment for indications of inflammation and cytotoxicity. Although histopathology was not performed on the animals used for genetic toxicity studies, an additional set of animals was removed from the 2‐year cancer bioassay for histopathological evaluation at the same time as the animals used for the genetic toxicity studies. No evidence of neoplastic lesions or nonneoplastic lesions, such as inflammation or necrosis was observed in the brains or livers of these animals, which could be attributable to RFR exposure (NTP 2018a; 2018b). Furthermore, RFR‐induced inflammation and necrosis were not observed in the brains or livers of rats or mice at the end of the 2‐year cancer bioassay (NTP 2018a; 2018b).

The NTP bioassay was designed to evaluate nonthermal effects of cell phone RFR exposure, which meant that body temperature could not change more than 1°C under our exposure conditions. To meet that requirement, pilot studies conducted to establish acceptable SARs for the bioassay indicated that no body temperature increases over 1°C would be expected in rats (including pregnant rats) or mice at exposures up to 6.0 or 10.0 W/kg, respectively (Wyde et al. 2018). Therefore, we consider it unlikely that thermal effects were a confounding factor for our genetic toxicity tests, although more work in general is needed to clarify the thermal effects of RFR on different tissues, and the degree to which increases in body or tissue temperature affect genomic integrity. Few studies have closely examined the relationship between increased body temperature and induction of DNA damage in mice, and there is almost no information on this relationship in rats. In one study in which the body temperatures of mice were closely monitored, an increase of ~2°C was required before increases in micronuclei were detected (Asanami and Shimono 1997).

Little is known about the mechanism by which RFR could induce DNA damage in the absence of heating. Unlike ionizing radiation or ultraviolet light, the radiation emitted by cell phones is not sufficiently energetic, by several orders of magnitude, to directly damage macromolecules (IARC Working Group on the Evaluation of Carcinogenic Risks to Humans 2013). Calculations by physicists and engineers suggest that RFR would not have an appreciable effect on biological systems at nonthermal levels of exposure, primarily due to the damping effects of water molecules (Adair 2002; 2003; Sheppard et al. 2008; IARC Working Group on the Evaluation of Carcinogenic Risks to Humans 2013). However, our results and the results of other experiments suggest that nonthermal exposure of cells or whole organisms to RFR may result in measurable genotoxic effects, despite varied and weak responses across studies overall (Brusick et al. 1998; Ruediger 2009; Verschaeve et al. 2010). Induction of oxygen radicals or interference with DNA repair processes has been proposed as possible mechanisms by which RFR could cause DNA damage (Ruediger 2009; Yakymenko et al. 2015).

NTP Technical Reports on the results of the 2‐year cancer bioassay for exposure to RFR for rats (TR 595) and mice (TR 596) were finalized, peer reviewed, and made publicly available in 2018. The NTP concluded that results demonstrated clear evidence of carcinogenic activity of cell phone RFR (both modulations) based on incidences of malignant schwannomas of the heart in male rats. Malignant gliomas in the brain were also observed in male rats exposed to cell phone RFR and were considered to be related to exposure. Female rats exhibited malignant schwannomas of the heart and malignant gliomas, but incidences of these tumors were considered equivocal. The observation that cell phone RFR affects heart and brain tissue in Sprague Dawley rats after long‐term exposure was replicated in a similar study (that used only the GSM modulation) by the Ramazzini Institute (Falcioni et al. 2018). The gliomas and schwannomas observed in rats are similar to the tumor types reported in some epidemiology studies to be associated with cell phone use. The NTP bioassay findings in mice, in which different organs were affected compared to rats, were considered equivocal. Notably, spontaneous and chemically induced brain tumors are rare in rats (Sills et al. 1999), and as of 2019, only 12 out of approximately 600 test articles have shown evidence of an increase in brain tumor incidence in rats in NTP bioassays.

The U.S. Federal Communications Commission has set a guideline limit for RFR requiring that mobile devices emit an SAR of less than of 1.6 W/kg as measured in a volume containing 1 g of tissue absorbing the signal. In contrast, animals in the NTP studies received whole‐body exposure to higher levels of RFR to identify potential target organs and to characterize toxicity. The highest exposure of 6 W/kg in rats and 10 W/kg in mice, for a total of 9 h 10 min a day (achieved by cycling for 10 min on, 10 min off over 18 h 20 min), produced higher exposures than experienced by humans under normal cellular phone use conditions. Thus, whether the findings in the NTP animal studies (eg, malignant gliomas in the brain and malignant schwannomas in the hearts of male rats; increased levels of DNA damage in hippocampal cells of male rats and the frontal cortex of male mice) indicate a potential for adverse health outcomes in humans remains a question. Because one of the most important questions prompted by our results concerns the mechanism(s) by which RFR might induce biological effects, follow‐up studies by the NTP to investigate mechanisms of genetic damage associated with RFR exposure are underway.

## AUTHOR CONTRIBUTIONS

K.L.W., M.E.W., G.E.K., R.R.T., and J.R.B. designed the study. S.L.S., G.E.K., K.R.S., and K.L.W. analyzed the data; S.L.S., G.E.K., K.R.S., and K.L.W. prepared the manuscript with input from C.A.H., K.G.S., R.R.T., and J.R.B. K.G.S and A.S.G. collected the tissue samples at IITRI and, together with J.W.W., conducted the genetic toxicity assays under the supervision of C.A.H. G.E.K. and K.R.S. performed statistical analyses of the data. M.E.W., M.D.S., R.R.T., C.A.H., and J.R.B. contributed important intellectual input to the study and to the manuscript, and all authors read and approved the manuscript.

## CONFLICT OF INTERESTS

The authors have no competing financial interests.

*Accepted by—*R. Preston

## Supporting information


**Figure S1** A – D. Consistency in scoring brain tissues meeting criteria for a positive call. (*A*) 100‐cell versus 150‐cell scoring method for male mouse frontal cortex, individual animal data. Each bar represents average % tail DNA per mouse. Error bars not shown for ease of comparison. (*B*) 100‐cell versus 150‐cell scoring method for male mouse frontal cortex tissue, dose group data. Each bar represents mean % tail DNA (+/‐ SE) per group. (*C*) 100‐cell versus 150‐cell scoring method for male rat hippocampus. Each bar represents average % tail DNA per rat. Error bars not shown for ease of comparison. (*D*) 100‐cell versus 150‐cell scoring method for male rat hippocampus, dose group data. Each bar represents mean % tail DNA (+/‐ SE) per group. Note that although including hedgehogs in the 150‐cell method increased the % tail DNA values, the overall pattern and results remained the same. Although male rat hippocampus was positive only for the 100‐cell data (Figures 1G, H), the overall difference between the two scoring methods was small.
**Figure S2** A – D. Representative comparison of the two comet assay scoring approaches in frontal cortex cells of male mice. Male mouse frontal cortex met the criteria for a positive call with both methods. The central horizontal bar indicates the mean; the vertical bar indicates the standard error of the mean. Each dot represents a % tail DNA value from one comet. Hedgehogs were excluded in the 100‐cell scoring method and were included in the 150‐cell method. Note: including hedgehogs results in capture of a wider range of % tail DNA.
**Figure S3 A – D**. Representative comparison of the two comet assay scoring approaches in hippocampal cells of male rats. Male rat hippocampus met the criteria for a positive call with the 100‐cell scoring approach. The central horizontal bar indicates the mean and the vertical bar indicates the standard error of the mean. Each dot represents a % tail DNA value from one comet. Hedgehogs were excluded in the 100‐cell scoring method and were included in the 150‐cell method. Note: including cells suspected to be hedgehogs at low scanning power results in capture of a wider range of % tail DNA. Rat tissues tended to show more hedgehogs than tissues from mice.

**Figure S4 A.** Shown are the mean % tail DNA data for each exposure for frontal cortex, hippocampus, cerebellum, liver, and blood tissues for male rats exposed to CDMA (top row) or GSM (bottom row) cell phone RFR modulations. The median value is marked with a + sign. Five animals are represented in each exposure group.
**Figure S4 B**. Shown are the mean % tail DNA data for each exposure for frontal cortex, hippocampus, cerebellum, liver, and blood tissues for female rats exposed to CDMA (top row) or GSM (bottom row) cell phone RFR modulations. The median value is marked with a + sign. Five rats are represented in each exposure except for sham, 1.5 W/kg GSM, and 3.0 W/kg GSM groups for hippocampal tissue, which are represented by 4 rats per group. One rat in the sham exposure group was omitted because it had a biologically implausible value of 56.1% tail DNA. One rat in the 1.5 W/kg GSM group and one rat in the 3.0 W/kg GSM group were omitted from analysis due to a labeling error during tissue collection.
**Figure S4 C**. Shown are the mean % tail DNA data for each exposure for frontal cortex, hippocampus, cerebellum, liver, and blood tissues for male mice exposed to CDMA (top row) or GSM (bottom row) cell phone RFR modulations. The median value is marked with a + sign. Five mice are represented in each exposure group.
**Figure S4 D**. Shown are the mean % tail DNA data for each exposure for frontal cortex, hippocampus, cerebellum, liver, and blood tissues for female mice exposed to CDMA (top row) or GSM (bottom row) cell phone RFR modulations. The median value is marked with a + sign. Five mice are represented in each exposure group.

**Table S1** Frequency of Micronuclei in Peripheral Blood Erythrocytes of Rats Following Exposure to CDMA‐ or GSM‐Modulated Cell Phone RFR for 19 Weeks^a^
**Table S2** Frequency of Micronuclei in Peripheral Blood Erythrocytes of Mice Following Exposure to CDMA‐ or GSM‐Modulated Cell Phone RFR for 14 Weeks^a^

## References

[em22343-bib-0001] Adair RK . 2002 Vibrational resonances in biological systems at microwave frequencies. Biophys J 82(3):1147–1152. 11867434 10.1016/S0006-3495(02)75473-8 PMC1301920

[em22343-bib-0002] Adair RK . 2003 Biophysical limits on athermal effects of RF and microwave radiation. Bioelectromagnetics 24(1):39–48. 12483664 10.1002/bem.10061

[em22343-bib-0091] Asanami S, Shimono K. 1997 High body temperature induces micronuclei in mouse bone marrow. *Mutat Res* 390(1‐2):79–83. 10.1016/s0165-1218(97)00002-5 9150755

[em22343-bib-0003] Brusick D , Albertini R , McRee D , Peterson D , Williams G , Hanawalt P , Preston J . 1998 Genotoxicity of radiofrequency radiation. DNA/Genetox Expert Panel. Environ Mol Mutagen 32(1):1–16. 9707093 10.1002/(sici)1098-2280(1998)32:1<1::aid-em1>3.0.co;2-q

[em22343-bib-0004] Capstick M , Kuster N , Kuehn S , Berdinas‐Torres V , Gong Y , Wilson P , Ladbury J , Koepke G , McCormick DL , Gauger J , et al. 2017 A radio frequency radiation exposure system for rodents based on reverberation chambers. IEEE Trans Electromagn Compat 59(4):1041–1052. 29217848 10.1109/TEMC.2017.2649885 PMC5714549

[em22343-bib-0005] Cardis E , Armstrong BK , Bowman JD , Giles GG , Hours M , Krewski D , McBride M , Parent ME , Sadetzki S , Woodward A , et al. 2011 Risk of brain tumours in relation to estimated RF dose from mobile phones: Results from five Interphone countries. Occup Environ Med 68(9):631–640. 21659469 10.1136/oemed-2011-100155 PMC3158328

[em22343-bib-0095] Falcioni L, Bua L, Tibaldi E, Lauriola M, De Angelis L, Gnudi F, Mandrioli D, Manservigi M, Manservisi F, Manzoli I, Menghetti I, Montella R, Panzacchi S, Sgargi D, Strollo V, Vornoli A, Belpoggi F. 2018 Report of final results regarding brain and heart tumors in Sprague‐Dawley rats exposed from prenatal life until natural death to mobile phone radiofrequency field representative of a 1.8 GHz GSM base station environmental emission. *Environ Res* 165:496–503. 10.1016/j.envres.2018.01.037 29530389

[em22343-bib-0006] Gong Y , Capstick M , Kuehn S , Wilson P , Ladbury J , Koepke G , McCormick DL , Melnick RL , Kuster N . 2017 Life‐time dosimetric assessment for mice and rats exposed in reverberation chambers of the 2‐year NTP cancer bioassay study on cell phone radiation. IEEE Trans Electromagn Compat 59(6):1798–1808. 29217849 10.1109/TEMC.2017.2665039 PMC5714545

[em22343-bib-0007] GSMA Intelligence . 2019 Definitive Data Analysis for the Mobile Industry, Global Data: Unique Mobile Subscribers, https://www.gsmaintelligence.com/, accessed June 6, 2019.

[em22343-bib-0008] Hardell L , Carlberg M . 2015 Mobile phone and cordless phone use and the risk for glioma—analysis of pooled case‐control studies in Sweden, 1997–2003 and 2007–2009. Pathophysiology 22(1):1–13. 25466607 10.1016/j.pathophys.2014.10.001

[em22343-bib-0009] Hardell L , Carlberg M , Hansson Mild K . 2011 Pooled analysis of case‐control studies on malignant brain tumours and the use of mobile and cordless phones including living and deceased subjects. Int J Oncol 38(5):1465–1474. 21331446 10.3892/ijo.2011.947

[em22343-bib-0010] Hartmann A , Agurell E , Beevers C , Brendler‐Schwaab S , Burlinson B , Clay P , Collins A , Smith A , Speit G , Thybaud V , et al. 2003 Recommendations for conducting the in vivo alkaline Comet assay. 4th International Comet Assay Workshop. Mutagenesis 18(1):45–51. 12473734 10.1093/mutage/18.1.45

[em22343-bib-0011] Hobbs CA , Chhabra RS , Recio L , Streicker M , Witt KL . 2012 Genotoxicity of styrene‐acrylonitrile trimer in brain, liver, and blood cells of weanling F344 rats. Environ Mol Mutagen 53(3):227–238. 22351108 10.1002/em.21680 PMC3520608

[em22343-bib-0012] IARC Working Group on the Evaluation of Carcinogenic Risks to Humans . 2013 Non‐ionizing radiation, Part 2: Radiofrequency electromagnetic fields. IARC Monogr Eval Carcinog Risks Hum 102(Pt 2):1–460. 24772662 PMC4780878

[em22343-bib-0013] International Telecommunication Union . 2017 ICT Development Report and Database, https://data.worldbank.org/indicator/IT.CEL.SETS?end=2017&start=1960&view=chart, accessed June 6, 2019. Geneva, Switzerland: The World Bank.

[em22343-bib-0014] INTERPHONE Study Group . 2010 Brain tumor risk in relation to mobile telephone use: Results of the INTERPHONE international case‐control study. Int J Epidemiol 39:675–694. 20483835 10.1093/ije/dyq079

[em22343-bib-0015] Kissling GE , Dertinger SD , Hayashi M , MacGregor JT . 2007 Sensitivity of the erythrocyte micronucleus assay: Dependence on number of cells scored and inter‐animal variability. Mutat Res 634(1–2):235–240. 17851117 10.1016/j.mrgentox.2007.07.010 PMC2133347

[em22343-bib-0016] Larjavaara S , Schuz J , Swerdlow A , Feychting M , Johansen C , Lagorio S , Tynes T , Klaeboe L , Tonjer SR , Blettner M , et al. 2011 Location of gliomas in relation to mobile telephone use: A case‐case and case‐specular analysis. Am J Epidemiol 174(1):2–11. 21610117 10.1093/aje/kwr071

[em22343-bib-0017] Lorenzo Y , Costa S , Collins AR , Azqueta A . 2013 The comet assay, DNA damage, DNA repair and cytotoxicity: Hedgehogs are not always dead. Mutagenesis 28(4):427–432. 23630247 10.1093/mutage/get018

[em22343-bib-0517] National Research Council (US) Institute for Laboratory Animal Research. 1996. Guide for the Care and Use of Laboratory Animals. Washington, DC: National Academies Press (US). 25121211

[em22343-bib-0018] National Toxicology Program (NTP) . 2018a Toxicology and Carcinogenesis Studies of GSM‐ and CDMA‐Modulated Cell Phone Radiofrequency Radiation at 900 MHz in Sprague Dawley Rats (Whole Body Exposure). Technical Report Series No. 595. USA: The National Toxicology Program. 10.22427/NTP-TR-595 PMC8039879 33562898

[em22343-bib-0019] National Toxicology Program (NTP) . 2018b Toxicology and Carcinogenesis Studies of GSM‐ and CDMA‐Modulated Cell Phone Radiofrequency Radiation at 1,900 MHz in B6C3F1/N Mice (Whole Body Exposure). Technical Report Series No. 596. USA: The National Toxicology Program. 10.22427/NTP-TR-596 PMC8040341 33562896

[em22343-bib-0020] OECD . 2014 Test No. 474: Mammalian Erythrocyte Micronucleus Test, OECD Guidelines for the Testing of Chemicals, Section 4. Paris: OECD Publishing.

[em22343-bib-0021] OECD . 2016 Test No. 489: in vivo Mammalian Alkaline Comet Assay, OECD Guidelines for the Testing of Chemicals, Section 4. Paris: OECD Publishing.

[em22343-bib-0022] Recio L , Kissling GE , Hobbs CA , Witt KL . 2012 Comparison of Comet assay dose‐response for ethyl methanesulfonate using freshly prepared versus cryopreserved tissues. Environ Mol Mutagen 53(2):101–113. 22069077 10.1002/em.20694

[em22343-bib-0023] Ruediger HW . 2009 Genotoxic effects of radiofrequency electromagnetic fields. Pathophysiology 16(2–3):89–102. 19285841 10.1016/j.pathophys.2008.11.004

[em22343-bib-0024] Rundell MS , Wagner ED , Plewa MJ . 2003 The comet assay: Genotoxic damage or nuclear fragmentation? Environ Mol Mutagen 42(2):61–67. 12929117 10.1002/em.10175

[em22343-bib-0025] Sato Y , Akiba S , Kubo O , Yamaguchi N . 2011 A case‐case study of mobile phone use and acoustic neuroma risk in Japan. Bioelectromagnetics 32(2):85–93. 21225885 10.1002/bem.20616

[em22343-bib-0026] Sheppard AR , Swicord ML , Balzano Q . 2008 Quantitative evaluations of mechanisms of radiofrequency interactions with biological molecules and processes. Health Phys 95(4):365–396. 18784511 10.1097/01.HP.0000319903.20660.37

[em22343-bib-0426] Sills RC, Hailey JR, Neal J, Boorman GA, Haseman JK, Melnick RL. 1999 Examination of low‐incidence brain tumor responses in F344 rats following chemical exposures in National Toxicology Program carcinogenicity studies. *Toxicol Pathol* 27(5):589–599. 10.1177/019262339902700513 10528639

[em22343-bib-0027] Speit G , Gminski R , Tauber R . 2013 Genotoxic effects of exposure to radiofrequency electromagnetic fields (RF‐EMF) in HL‐60 cells are not reproducible. Mutat Res 755(2):163–166. 23817106 10.1016/j.mrgentox.2013.06.014

[em22343-bib-0028] Verschaeve L , Juutilainen J , Lagroye I , Miyakoshi J , Saunders R , de Seze R , Tenforde T , van Rongen E , Veyret B , Xu Z . 2010 In vitro and in vivo genotoxicity of radiofrequency fields. Mutat Res 705(3):252–268. 20955816 10.1016/j.mrrev.2010.10.001

[em22343-bib-0029] Witt KL , Livanos E , Kissling GE , Torous DK , Caspary W , Tice RR , Recio L . 2008 Comparison of flow cytometry‐ and microscopy‐based methods for measuring micronucleated reticulocyte frequencies in rodents treated with nongenotoxic and genotoxic chemicals. Mutat Res 649(1–2):101–113. 17869571 10.1016/j.mrgentox.2007.08.004 PMC2234598

[em22343-bib-0030] Wyde ME , Horn TL , Capstick MH , Ladbury JM , Koepke G , Wilson PF , Kissling GE , Stout MD , Kuster N , Melnick RL , et al. 2018 Effect of cell phone radiofrequency radiation on body temperature in rodents: Pilot studies of the National Toxicology Program's reverberation chamber exposure system. Bioelectromagnetics 39(3):190–199. 29537695 10.1002/bem.22116

[em22343-bib-0031] Yakymenko I , Tsybulin O , Sidorik E , Henshel D , Kyrylenko O , Kyrylenko S . 2015 Oxidative mechanisms of biological activity of low‐intensity radiofrequency radiation. Electromagn Biol Med 35:1–16. 10.3109/15368378.2015.1043557 26151230

