# Supplementary material for: Evaluation of the genotoxicity of cell phone radiofrequency radiation in male and female rats and mice following subchronic exposure
Source: Environ Mol Mutagen. 2019 Nov 13;61(2):276–90. doi: 10.1002/em.22343 (PMC7027901; doi:10.1002/em.22343)
Supplement: Supplementary file 1 — Figure S1 A – D. Consistency in scoring brain tissues meeting criteria for a positive call. (A) 100‐cell versus 150‐cell scoring method for male mouse frontal cortex, individual animal data. Each bar represents average % tail DNA per mouse. Error bars not shown for ease of comparison. (B) 100‐cell versus 150‐cell scoring method for male mouse frontal cortex tissue, dose group data. Each bar represents mean % tail DNA (+/‐ SE) per group. (C) 100‐cell versus 150‐cell scoring method for male rat hippocampus. Each bar represents average % tail DNA per rat. Error bars not shown for ease of comparison. (D) 100‐cell versus 150‐cell scoring method for male rat hippocampus, dose group data. Each bar represents mean % tail DNA (+/‐ SE) per group. Note that although including hedgehogs in the 150‐cell method increased the % tail DNA values, the overall pattern and results remained the same. Although male rat hippocampus was positive only for the 100‐cell data (Figures 1G, H), the overall difference between the two scoring methods was small. Figure S2 A – D. Representative comparison of the two comet assay scoring approaches in frontal cortex cells of male mice. Male mouse frontal cortex met the criteria for a positive call with both methods. The central horizontal bar indicates the mean; the vertical bar indicates the standard error of the mean. Each dot represents a % tail DNA value from one comet. Hedgehogs were excluded in the 100‐cell scoring method and were included in the 150‐cell method. Note: including hedgehogs results in capture of a wider range of % tail DNA. Figure S3 A – D. Representative comparison of the two comet assay scoring approaches in hippocampal cells of male rats. Male rat hippocampus met the criteria for a positive call with the 100‐cell scoring approach. The central horizontal bar indicates the mean and the vertical bar indicates the standard error of the mean. Each dot represents a % tail DNA value from one comet. Hedgehogs were excluded in the 100‐c [file EM-61-276-s001.docx]

**Supporting Information Figure 1A-D**

**Male Mouse Frontal Cortex**

**Mean % Tail DNA**


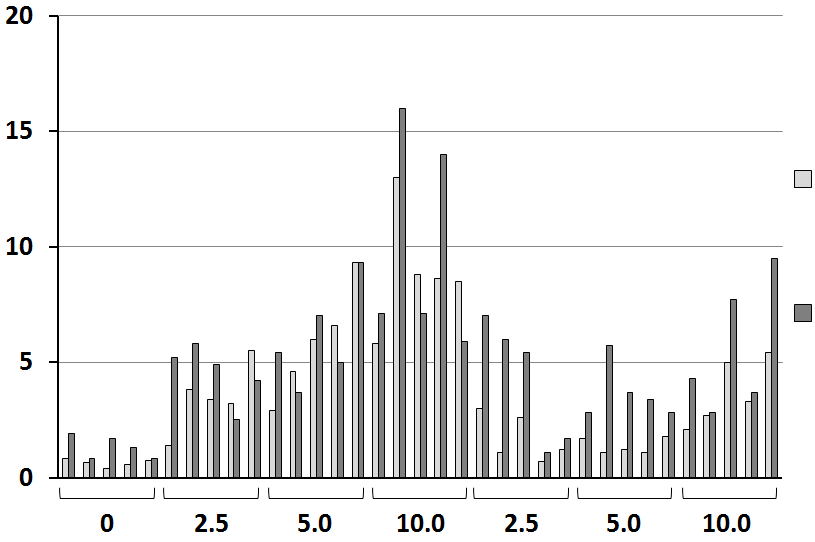


**Sham**

**CDMA (W/kg)**

**GSM (W/kg)**

**100-cell**

**scoring**

**150-cell**

**scoring**

**A**


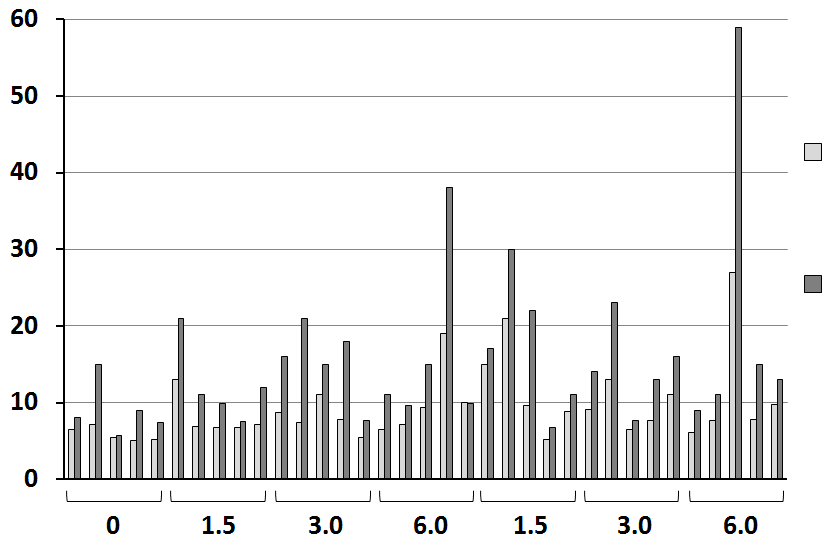


**Mean % Tail DNA**

**Male Rat Hippocampus**

**Sham**

**CDMA (W/kg)**

**GSM (W/kg)**

**100-cell**

**scoring**

**150-cell**

**scoring**

**C**

Figure S1A – D. Consistency in scoring brain tissues meeting criteria for a positive call. (*A*) 100-cell versus 150-cell scoring method for male mouse frontal cortex, individual animal data. Each bar represents average % tail DNA per mouse. Error bars not shown for ease of comparison. (*B*) 100-cell versus 150-cell scoring method for male mouse frontal cortex tissue, dose group data. Each bar represents mean % tail DNA (+/- SE) per group. (*C*) 100-cell versus 150-cell scoring method for male rat hippocampus. Each bar represents average % tail DNA per rat. Error bars not shown for ease of comparison. (*D*) 100-cell versus 150-cell scoring method for male rat hippocampus, dose group data. Each bar represents mean % tail DNA (+/- SE) per group. Note that although including hedgehogs in the 150-cell method increased the % tail DNA values, the overall pattern and results remained the same. Although male rat hippocampus was positive only for the 100-cell data (Figures 1G, H), the overall difference between the two scoring methods was small.

**B**

**Mean % Tail DNA**

**Male Mouse Frontal Cortex**

**D**

**Mean % Tail DNA**

**Male Rat Hippocampus**

Supporting Information Figure 2A-D


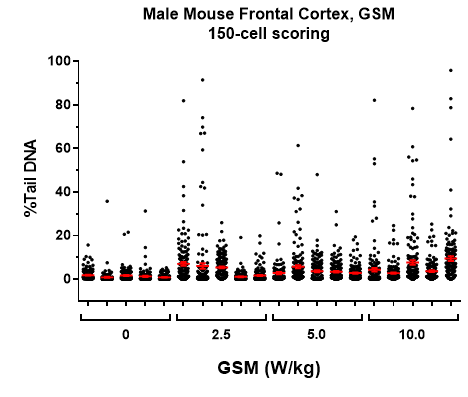


Figure S2A – D. Representative comparison of the two comet assay scoring approaches in frontal cortex cells of male mice. Male mouse frontal cortex met the criteria for a positive call with both methods. The central horizontal bar indicates the mean; the vertical bar indicates the standard error of the mean. Each dot represents a % tail DNA value from one comet. Hedgehogs were excluded in the 100-cell scoring method and were included in the 150-cell method. Note: including hedgehogs results in capture of a wider range of % tail DNA.

Supporting Information Figure 3A-D

**A**

**B**

**C**

**D**

Figure S3A – D. Representative comparison of the two comet assay scoring approaches in hippocampal cells of male rats. Male rat hippocampus met the criteria for a positive call with the 100-cell scoring approach. The central horizontal bar indicates the mean and the vertical bar indicates the standard error of the mean. Each dot represents a % tail DNA value from one comet. Hedgehogs were excluded in the 100-cell scoring method and were included in the 150-cell method. Note: including cells suspected to be hedgehogs at low scanning power results in capture of a wider range of % tail DNA. Rat tissues tended to show more hedgehogs than tissues from mice.
