# Supplementary material for: Evaluation of the genotoxicity of cell phone radiofrequency radiation in male and female rats and mice following subchronic exposure
Source: Environ Mol Mutagen. 2019 Nov 13;61(2):276–90. doi: 10.1002/em.22343 (PMC7027901; doi:10.1002/em.22343)

Supporting Information Figure 4A


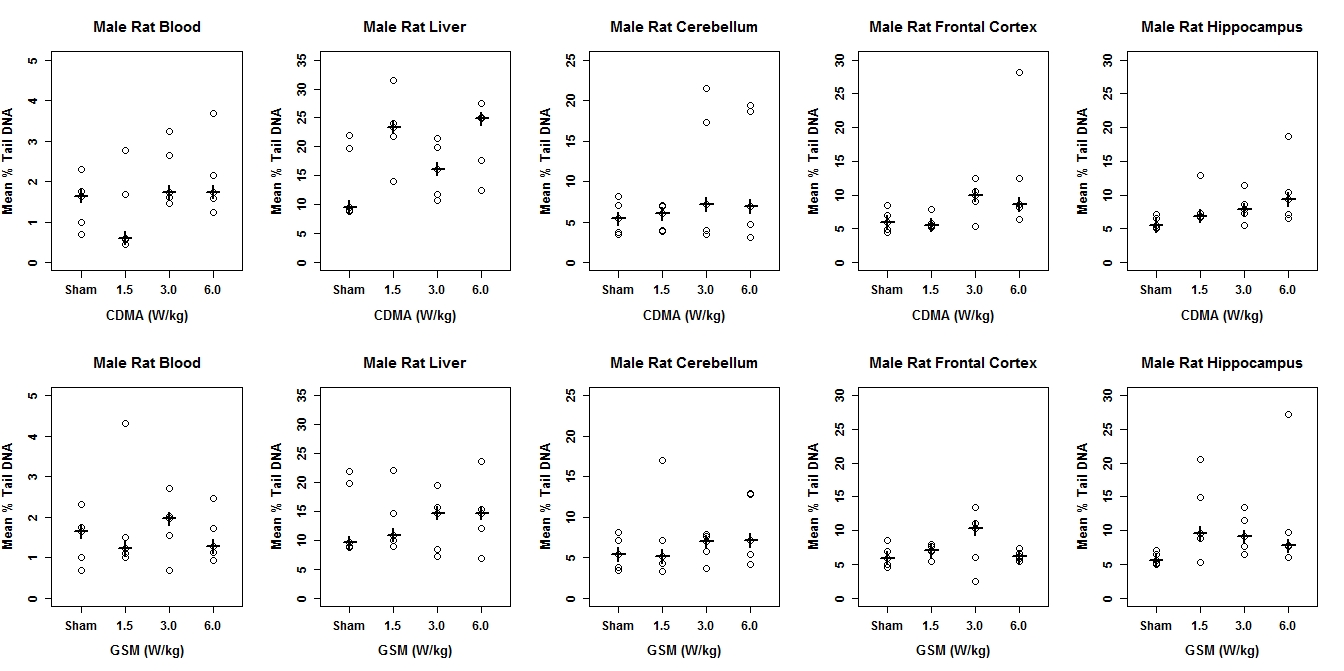


Figure 4A. Shown are the mean % tail DNA data for each exposure for frontal cortex, hippocampus, cerebellum, liver, and blood tissues for male rats exposed to CDMA (top row) or GSM (bottom row) cell phone RFR modulations. The median value is marked with a + sign. Five animals are represented in each exposure group.


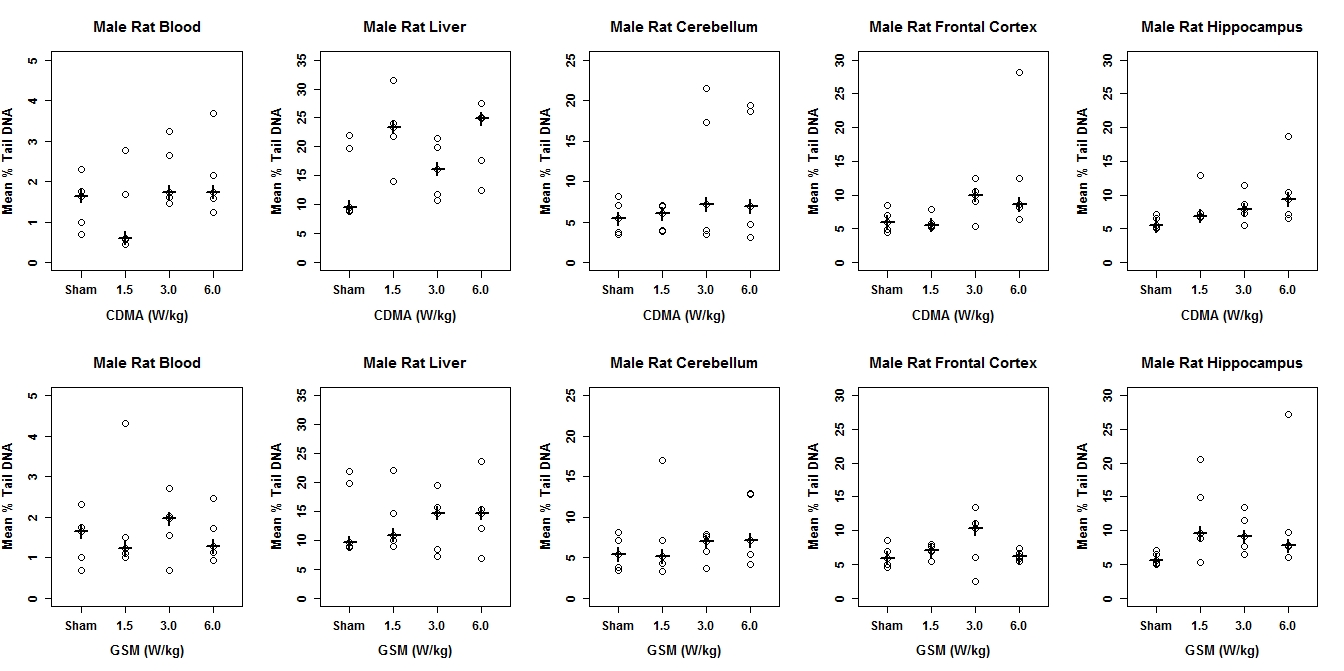

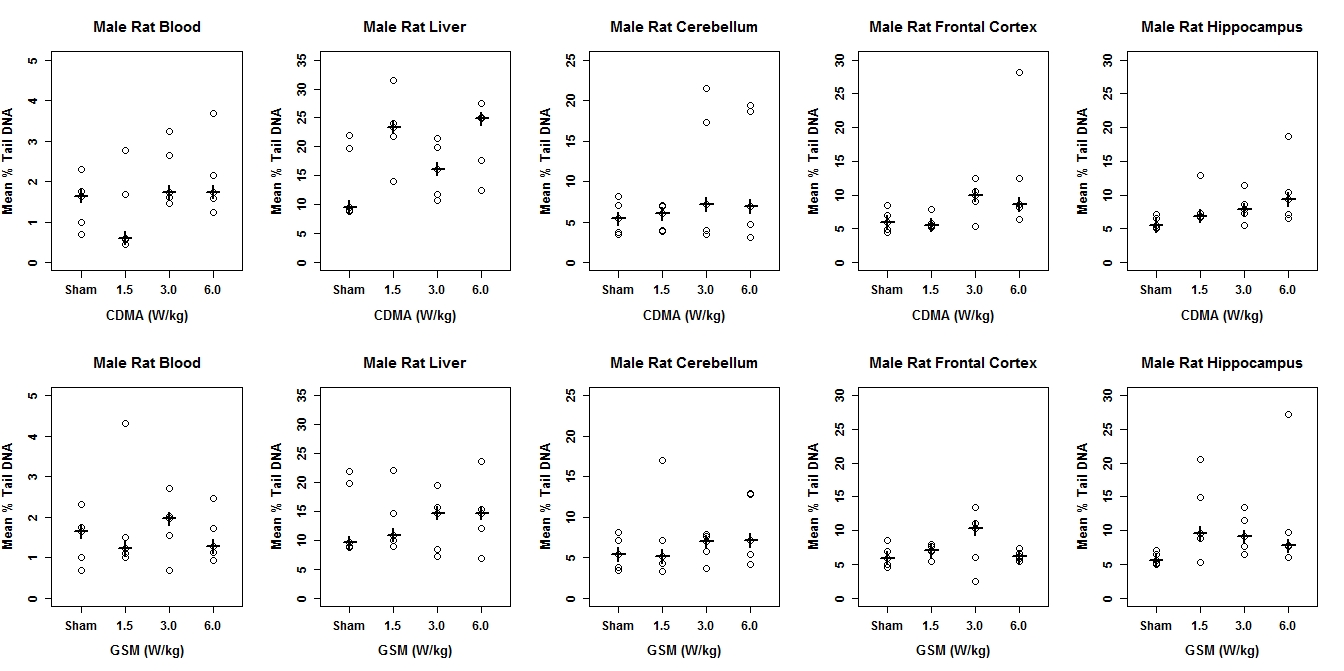

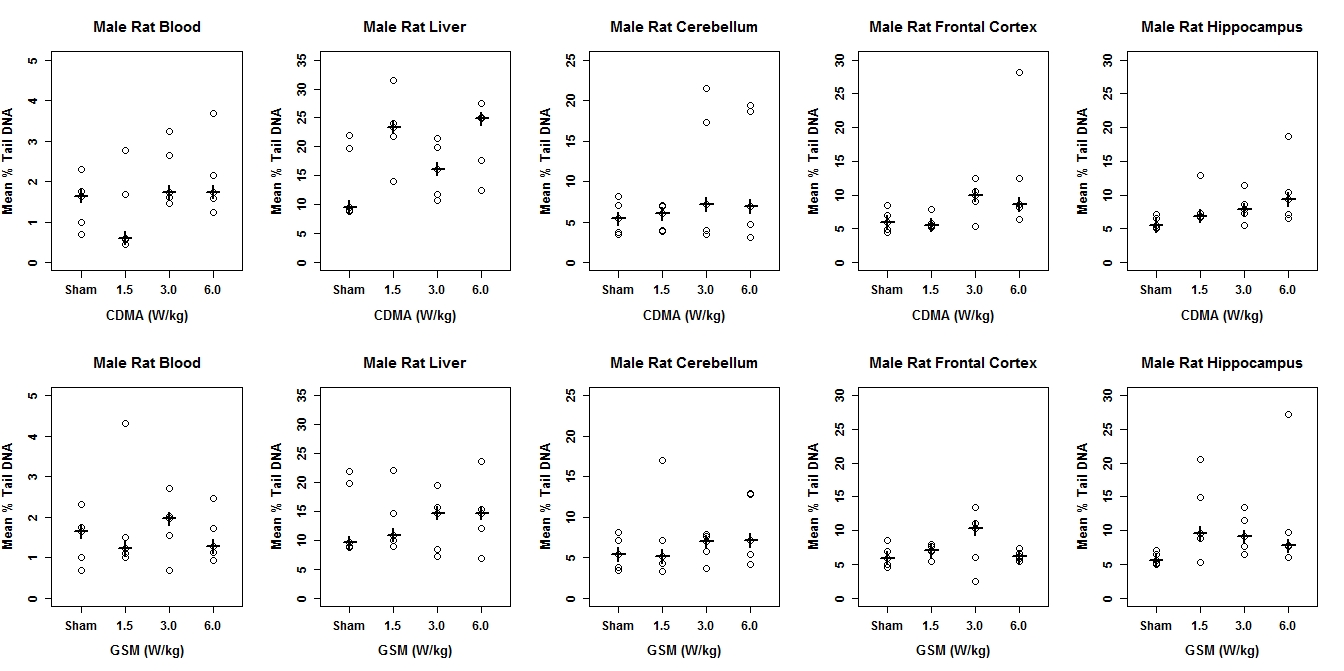

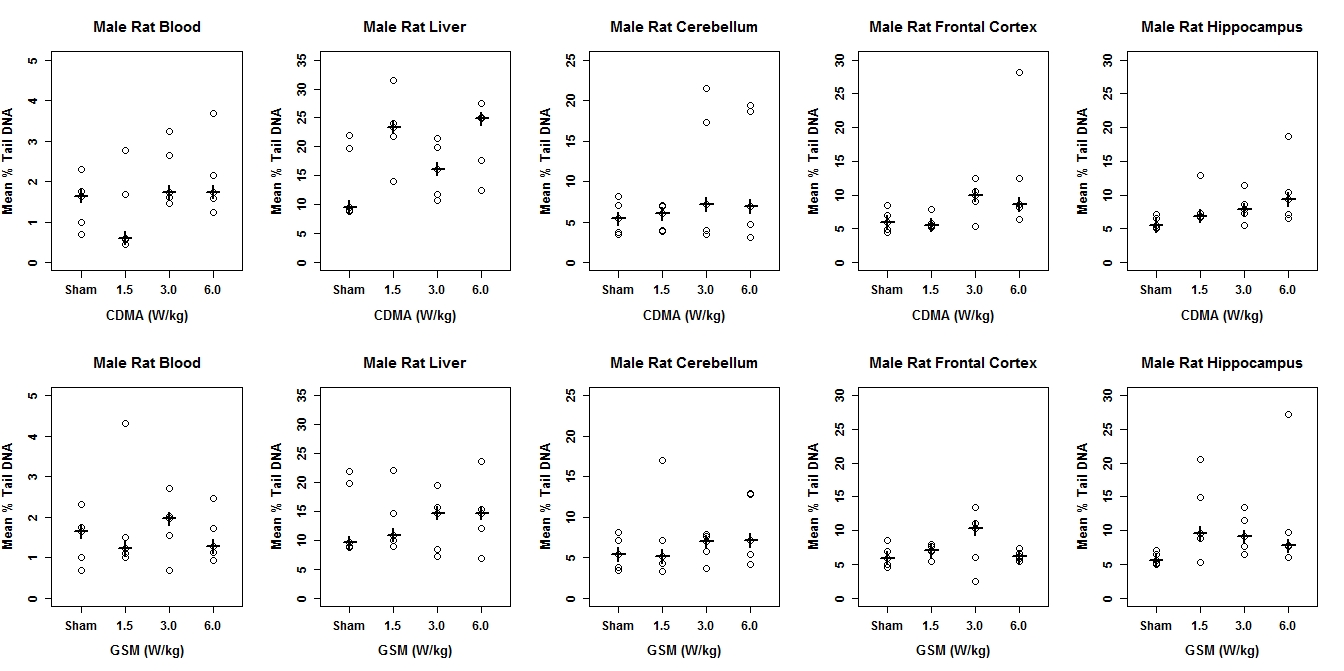


Supporting Information Figure 4B


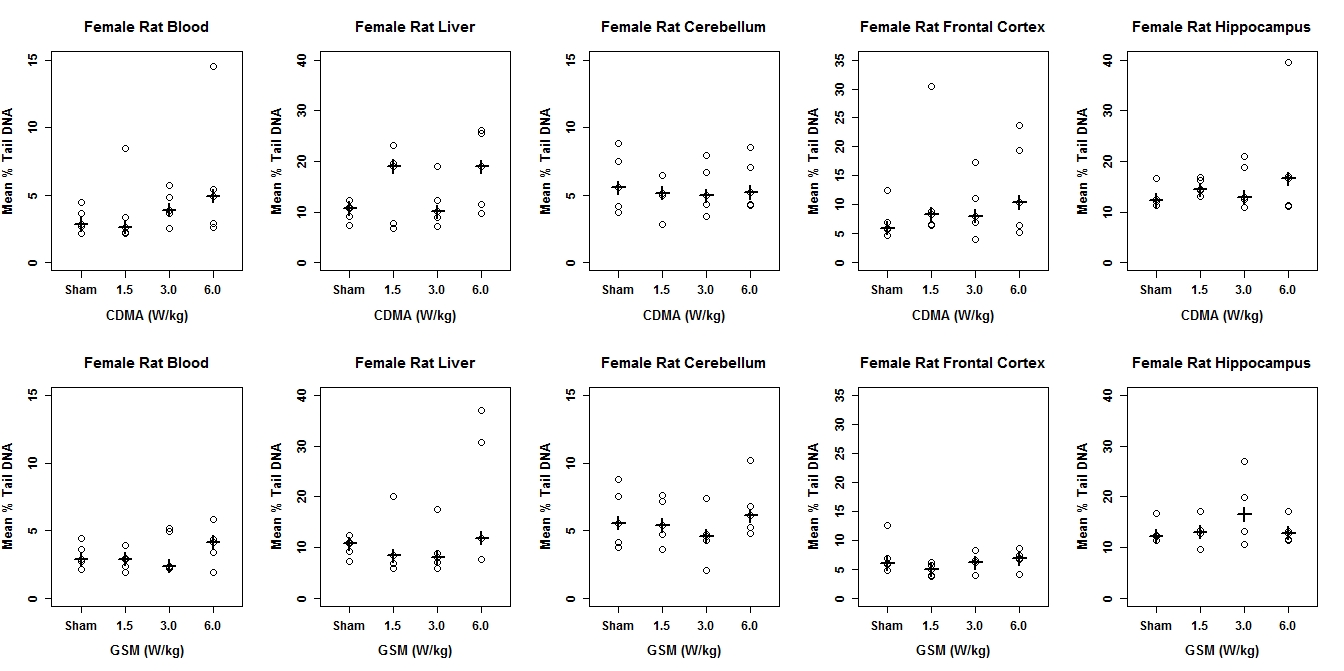


Figure 4B. Shown are the mean % tail DNA data for each exposure for frontal cortex, hippocampus, cerebellum, liver, and blood tissues for female rats exposed to CDMA (top row) or GSM (bottom row) cell phone RFR modulations. The median value is marked with a + sign. Five rats are represented in each exposure except for sham, 1.5 W/kg GSM, and 3.0 W/kg GSM groups for hippocampal tissue, which are represented by 4 rats per group. One rat in the sham exposure group was omitted because it had a biologically implausible value of 56.1% tail DNA. One rat in the 1.5 W/kg GSM group and one rat in the 3.0 W/kg GSM group were omitted from analysis due to a labeling error during tissue collection.


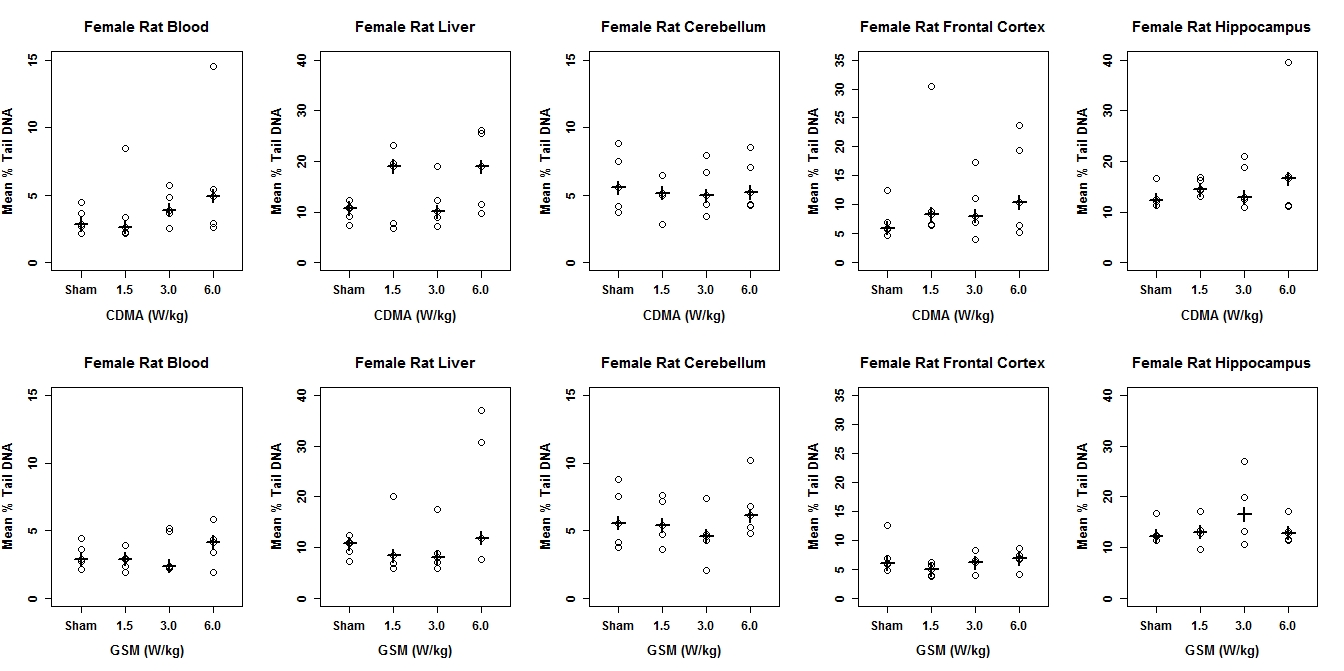

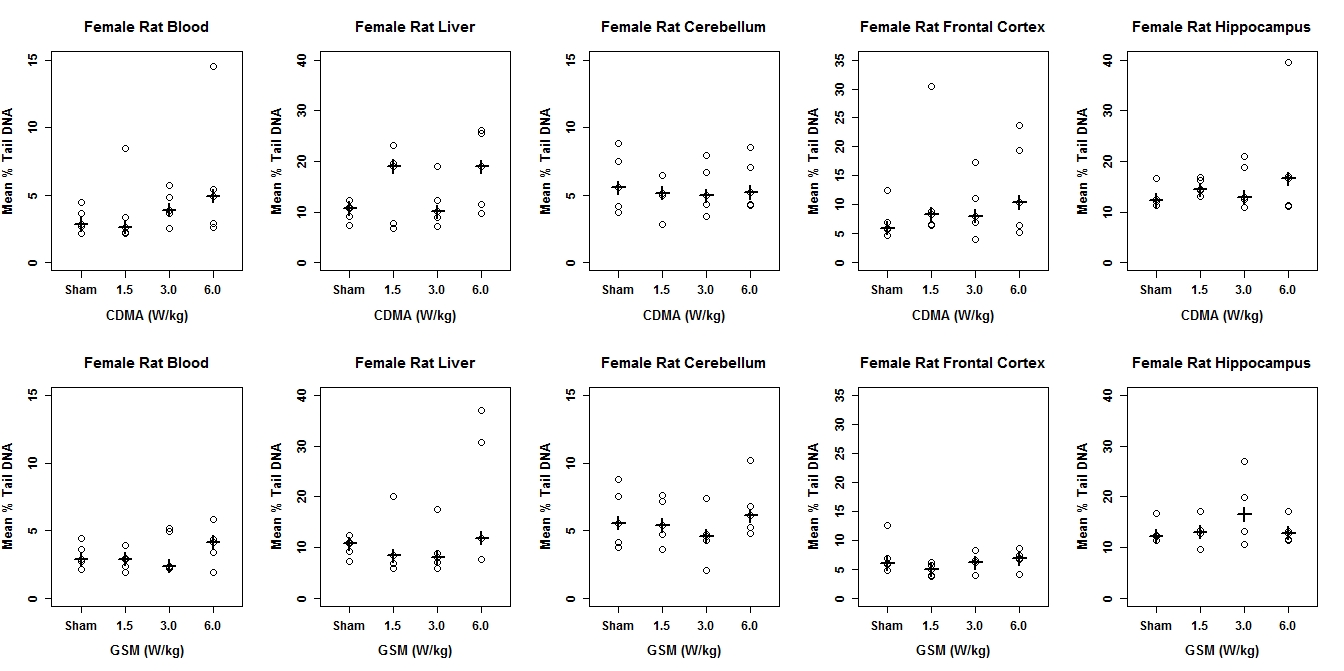

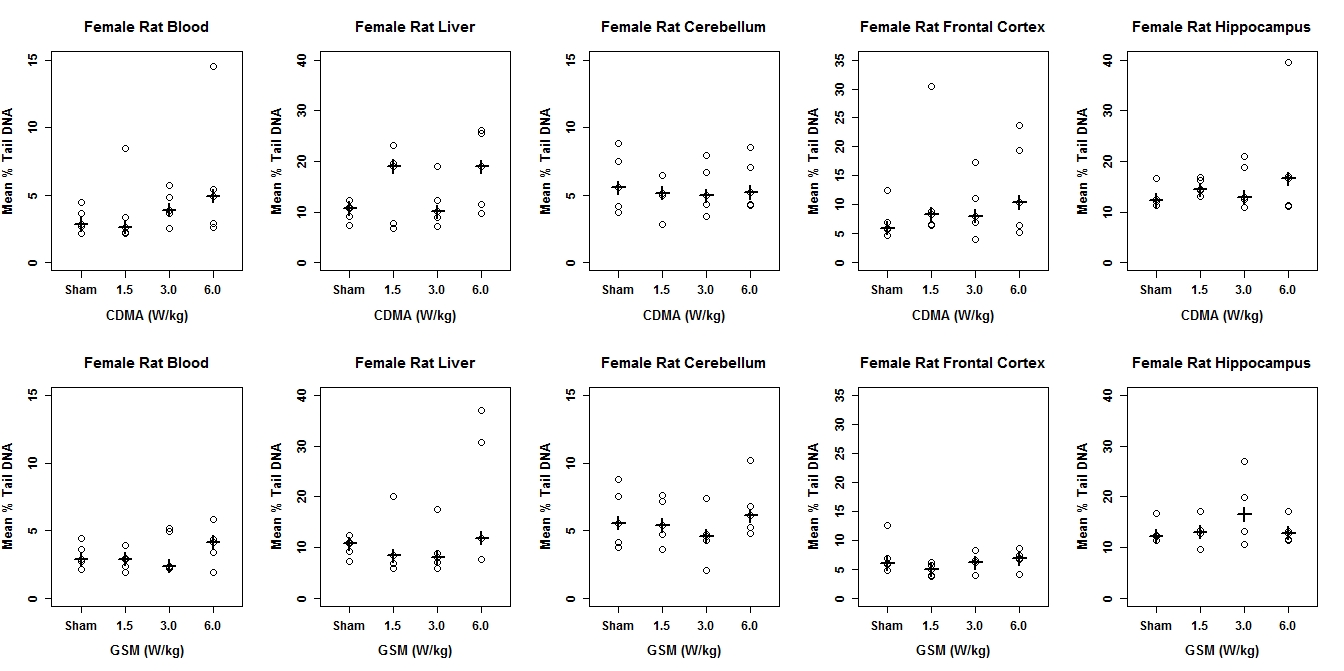

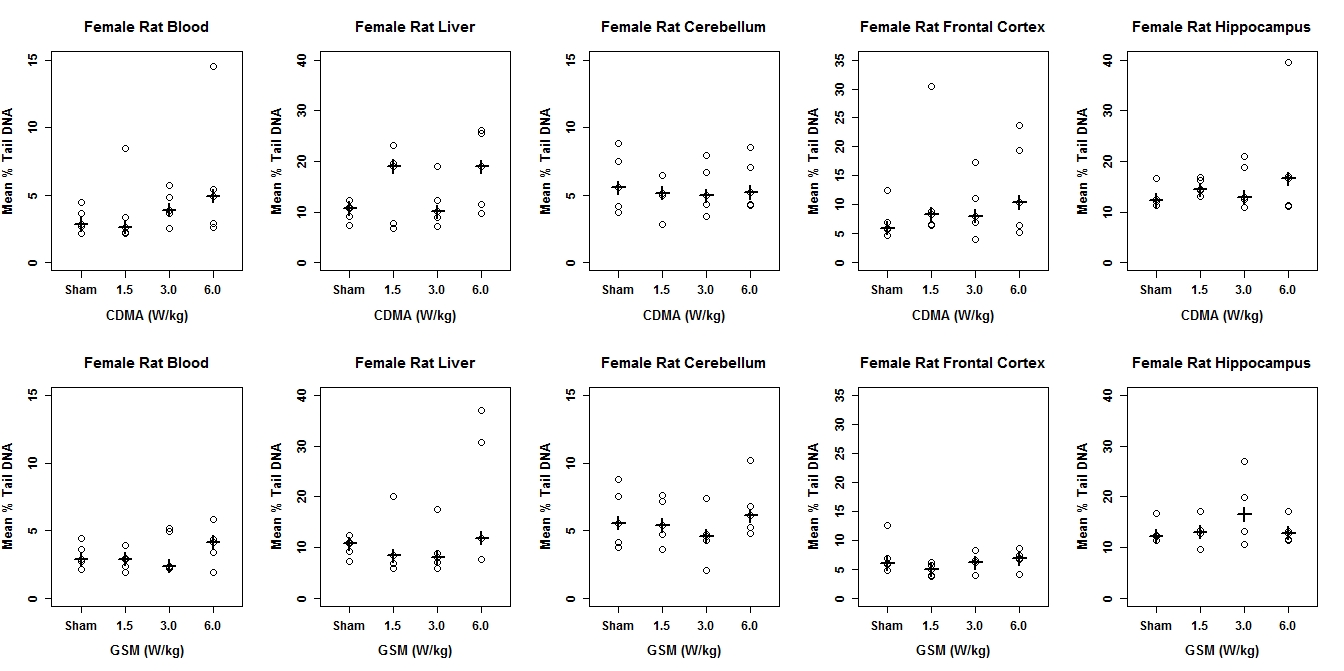


Supporting Information Figure 4C


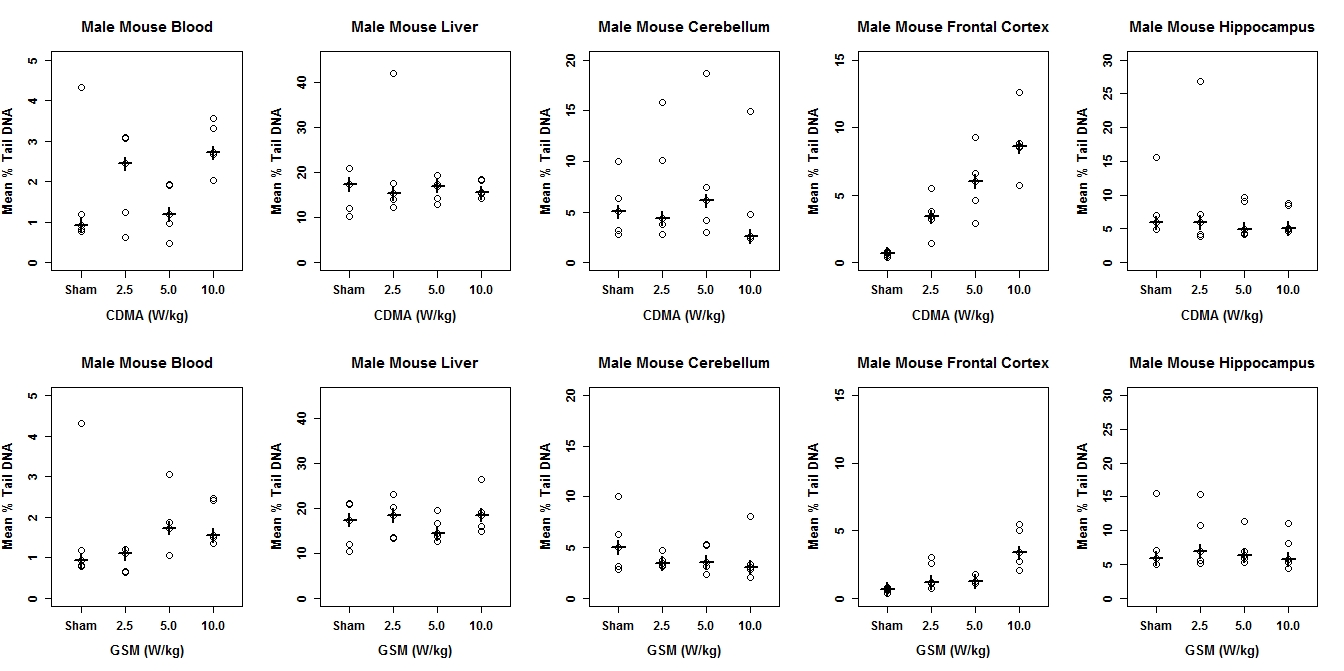


Figure 4C. Shown are the mean % tail DNA data for each exposure for frontal cortex, hippocampus, cerebellum, liver, and blood tissues for male mice exposed to CDMA (top row) or GSM (bottom row) cell phone RFR modulations. The median value is marked with a + sign. Five mice are represented in each exposure group.


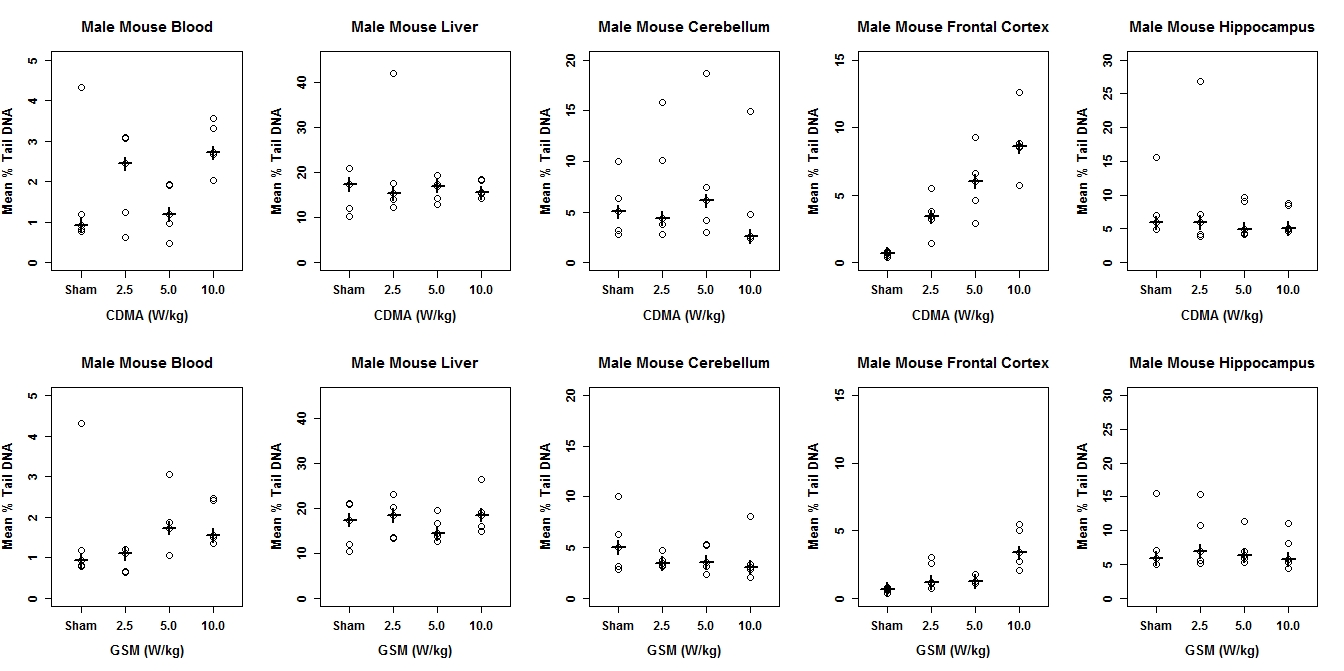

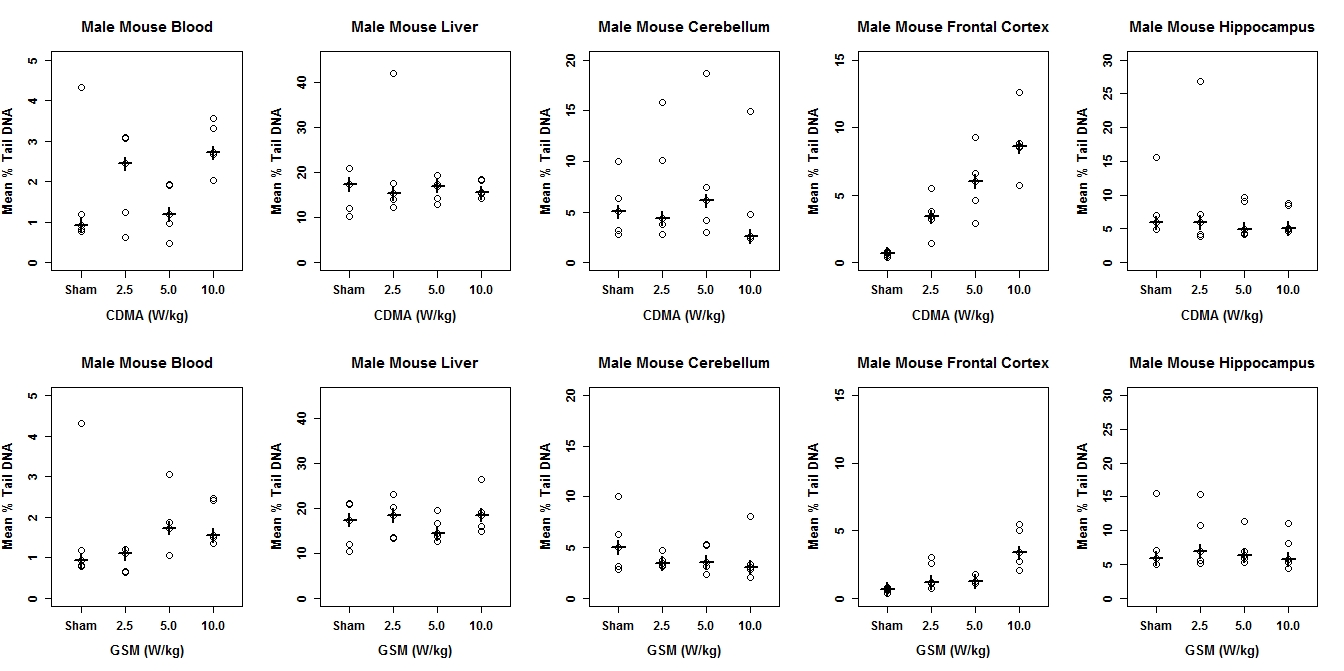

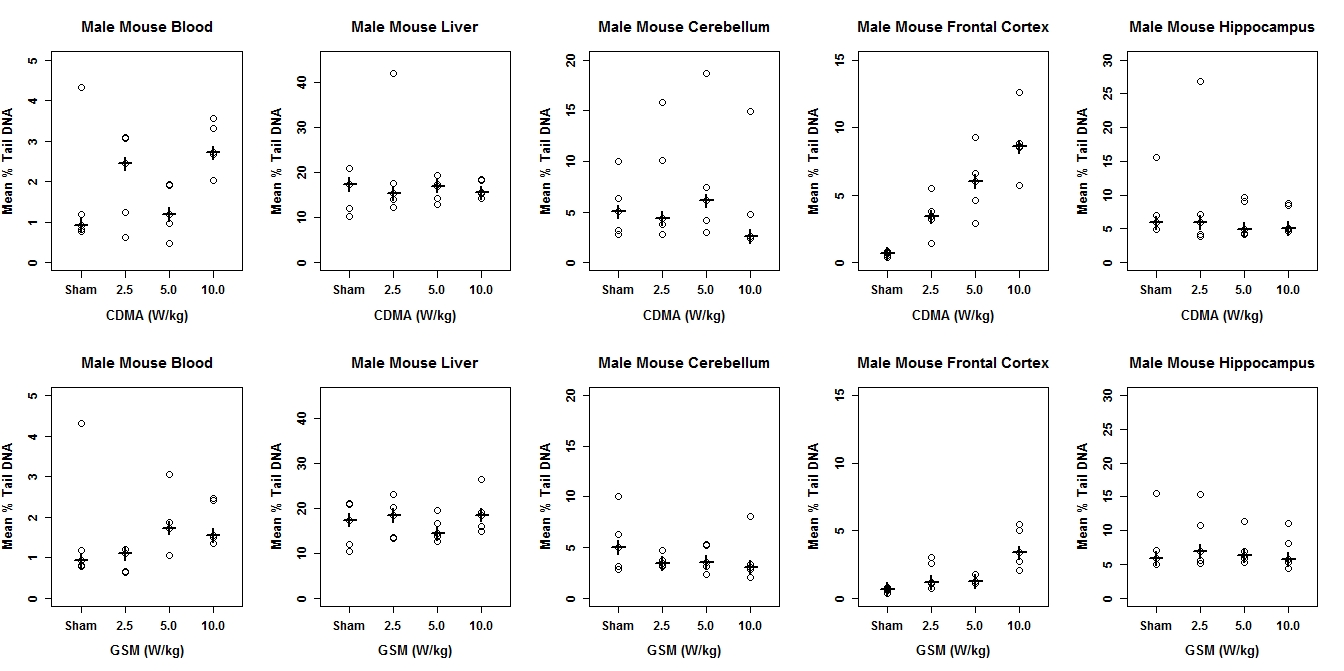

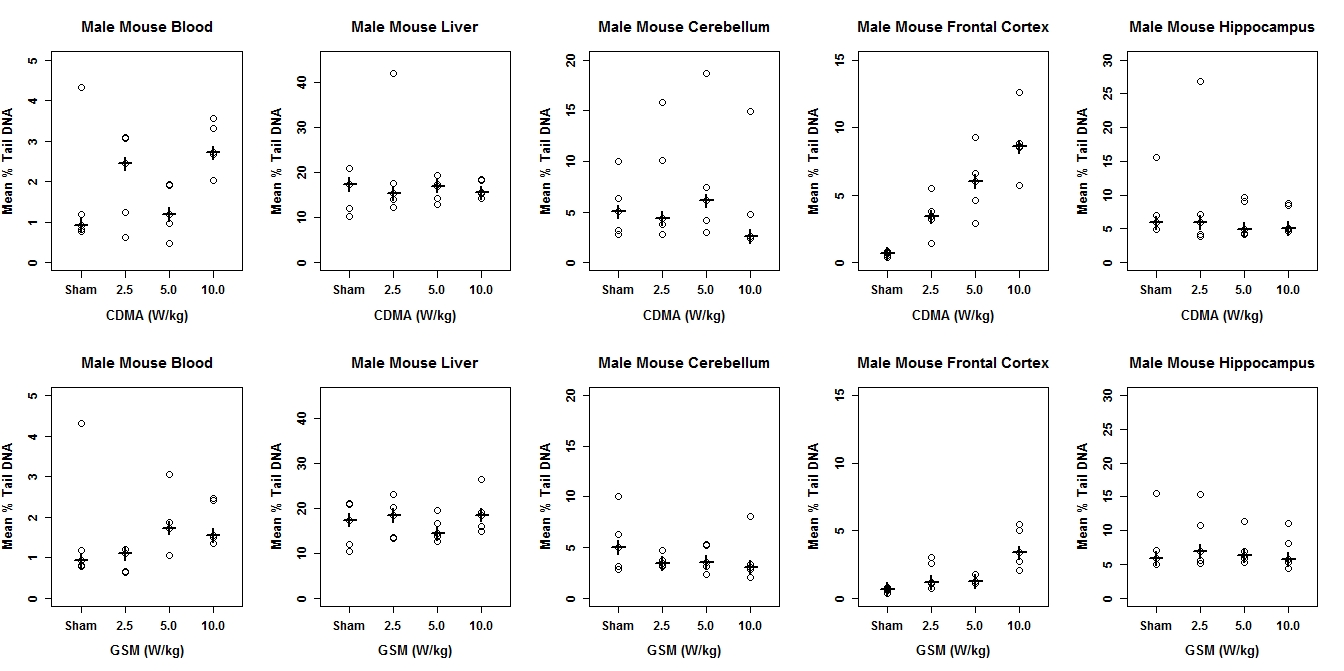


Supporting Information Figure 4D


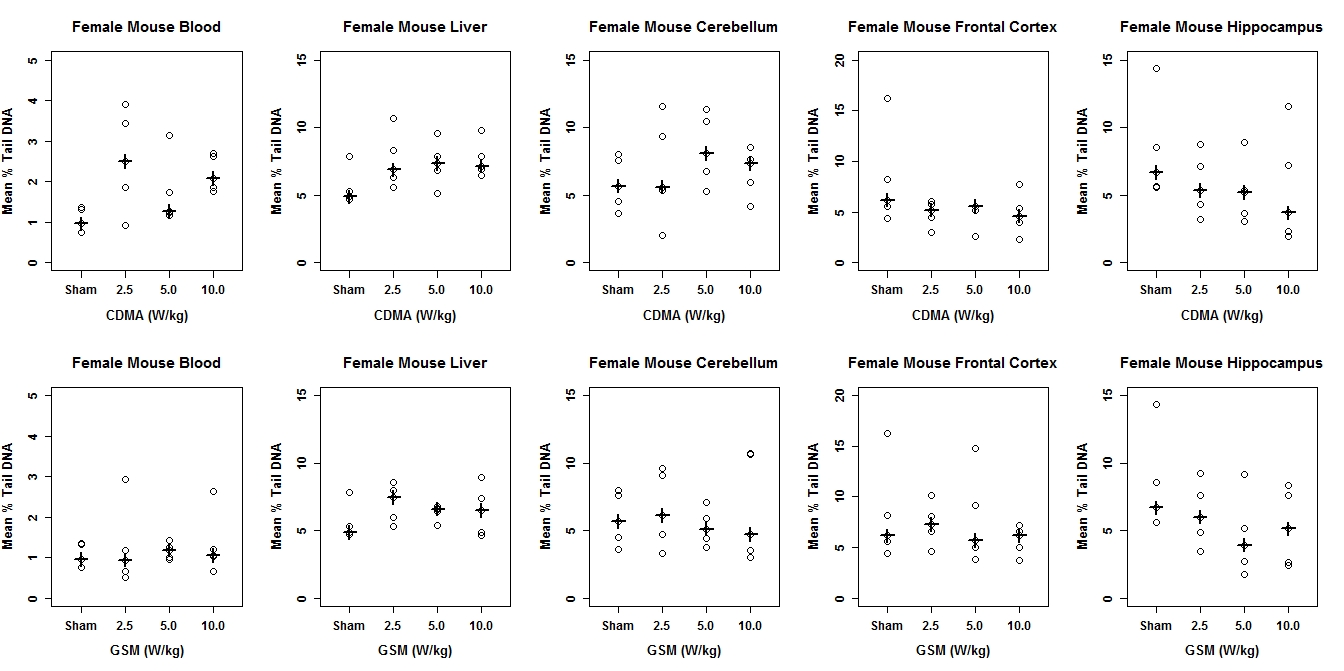


Figure 4D. Shown are the mean % tail DNA data for each exposure for frontal cortex, hippocampus, cerebellum, liver, and blood tissues for female mice exposed to CDMA (top row) or GSM (bottom row) cell phone RFR modulations. The median value is marked with a + sign. Five mice are represented in each exposure group.


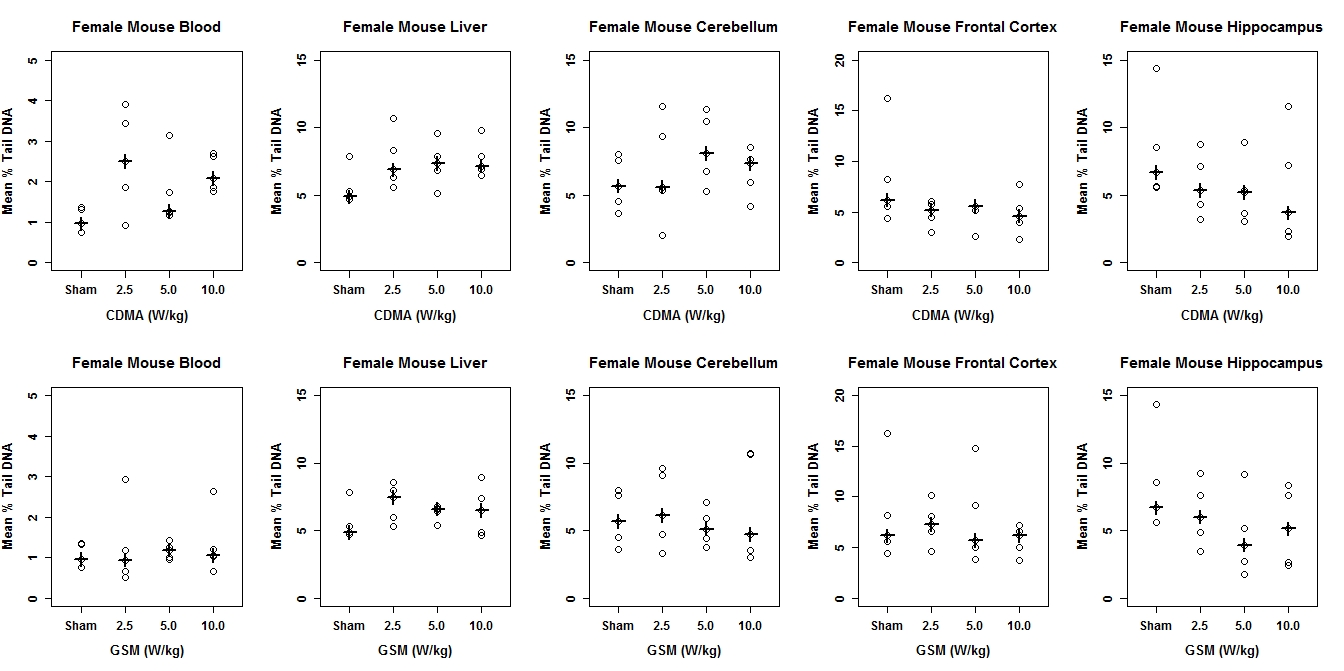

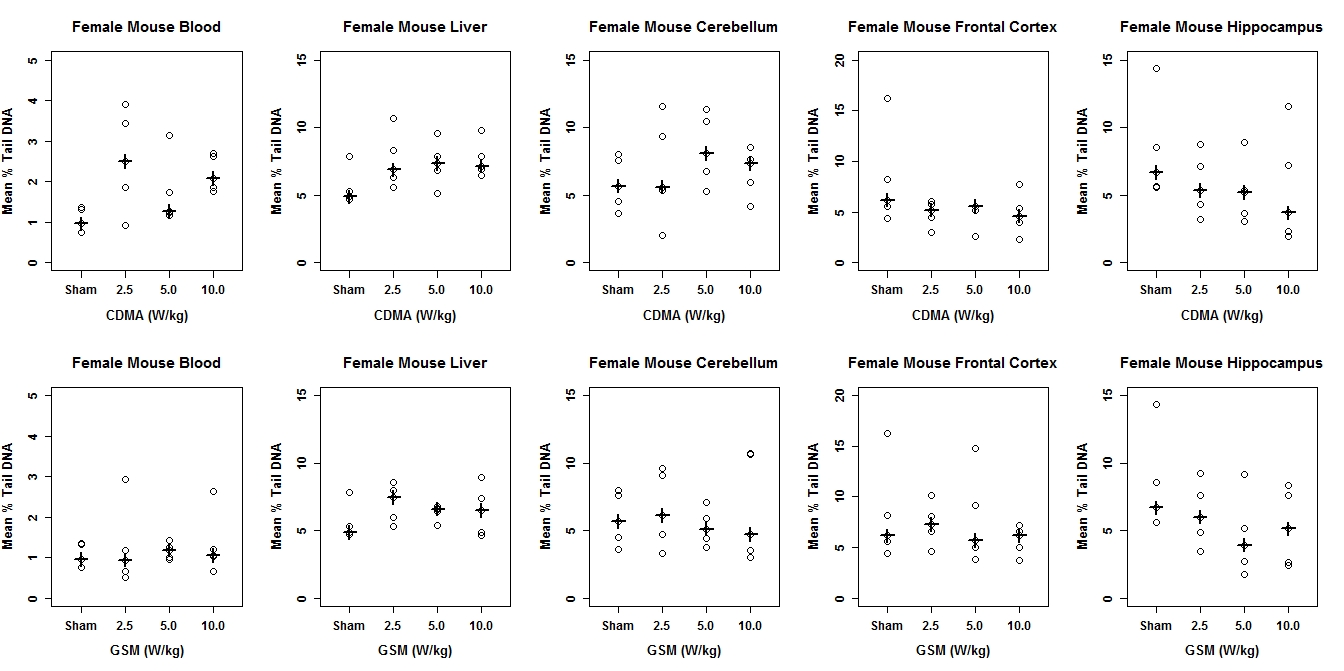

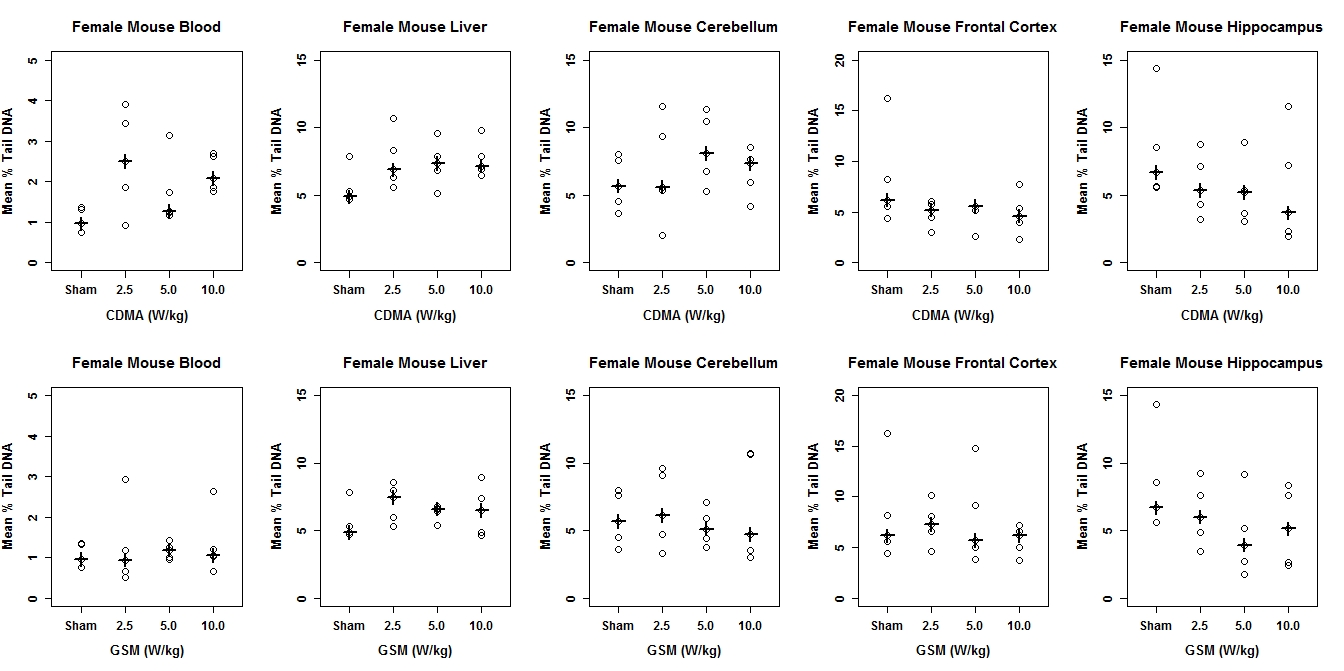

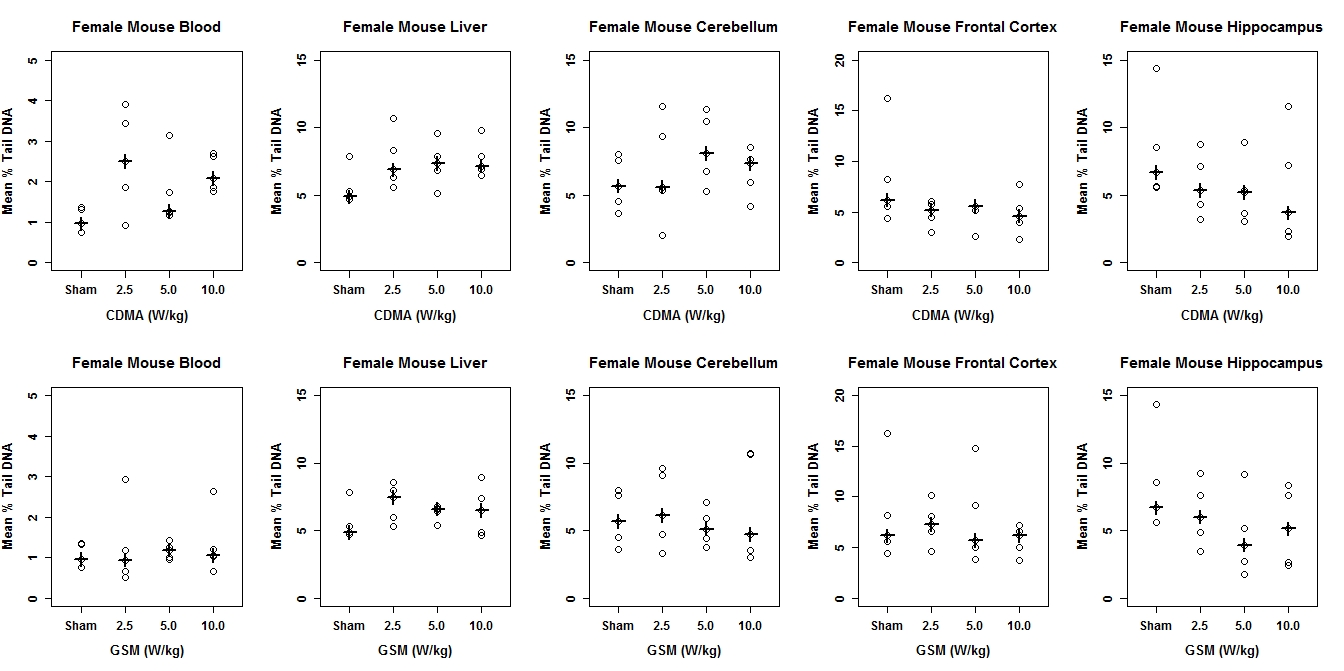

Supplement: Supplementary file 2 — Figure S4 A. Shown are the mean % tail DNA data for each exposure for frontal cortex, hippocampus, cerebellum, liver, and blood tissues for male rats exposed to CDMA (top row) or GSM (bottom row) cell phone RFR modulations. The median value is marked with a + sign. Five animals are represented in each exposure group. Figure S4 B. Shown are the mean % tail DNA data for each exposure for frontal cortex, hippocampus, cerebellum, liver, and blood tissues for female rats exposed to CDMA (top row) or GSM (bottom row) cell phone RFR modulations. The median value is marked with a + sign. Five rats are represented in each exposure except for sham, 1.5 W/kg GSM, and 3.0 W/kg GSM groups for hippocampal tissue, which are represented by 4 rats per group. One rat in the sham exposure group was omitted because it had a biologically implausible value of 56.1% tail DNA. One rat in the 1.5 W/kg GSM group and one rat in the 3.0 W/kg GSM group were omitted from analysis due to a labeling error during tissue collection. Figure S4 C. Shown are the mean % tail DNA data for each exposure for frontal cortex, hippocampus, cerebellum, liver, and blood tissues for male mice exposed to CDMA (top row) or GSM (bottom row) cell phone RFR modulations. The median value is marked with a + sign. Five mice are represented in each exposure group. Figure S4 D. Shown are the mean % tail DNA data for each exposure for frontal cortex, hippocampus, cerebellum, liver, and blood tissues for female mice exposed to CDMA (top row) or GSM (bottom row) cell phone RFR modulations. The median value is marked with a + sign. Five mice are represented in each exposure group. [file EM-61-276-s002.docx]
