# Supplementary material for: Evaluation of the genotoxicity of cell phone radiofrequency radiation in male and female rats and mice following subchronic exposure
Source: Environ Mol Mutagen. 2019 Nov 13;61(2):276–90. doi: 10.1002/em.22343 (PMC7027901; doi:10.1002/em.22343)
Supplement: Supplementary file 3 — Table S1 Frequency of Micronuclei in Peripheral Blood Erythrocytes of Rats Following Exposure to CDMA‐ or GSM‐Modulated Cell Phone RFR for 19 Weeksa Table S2 Frequency of Micronuclei in Peripheral Blood Erythrocytes of Mice Following Exposure to CDMA‐ or GSM‐Modulated Cell Phone RFR for 14 Weeksa [file EM-61-276-s003.docx]

**Supporting Information Table I**

###### **Frequency of Micronuclei in Peripheral Blood Erythrocytes of Rats Following Exposure to CDMA- or GSM-Modulated Cell Phone RFR for 19 Weeksa**

|  |  |  |  |  |  |  |  |  |
| --- | --- | --- | --- | --- | --- | --- | --- | --- |
|  |  |  |  |  |  |  |  |  |
|  | **Dose (W/kg)** | **Number of Rats with Erythrocytes Scored** | **Micronucleated RETs/**  **1,000 RETs**b | **P Value**c | **Micronucleated Mature Erythrocytes/**  **1,000 Mature Erythrocytes**b | **P Value**c | **RETs**b **(%)** | **P Value**c |
| **Male** |  |  |  |  |  |  |  |  |
| Sham Controld | 0 | 5 | 0.84 ± 0.10 |  | 0.33 ± 0.11 |  | 0.95 ± 0.05 |  |
| CDMA | 1.5 | 5 | 0.56 ± 0.02 | 0.989 | 0.12 ± 0.02 | 1.000 | 0.83 ± 0.07 | 0.588 |
|  | 3 | 5 | 0.55 ± 0.06 | 0.997 | 0.13 ± 0.02 | 1.000 | 0.88 ± 0.05 | 0.700 |
|  | 6 | 5 | 0.43 ± 0.07 | 0.998 | 0.13 ± 0.05 | 1.000 | 0.99 ± 0.07 | 0.741 |
|  |  |  | P=0.999e |  | P=0.970 |  | P=0.389 |  |
| GSM | 1.5 | 5 | 0.61 ± 0.11 | 0.920 | 0.14 ± 0.04 | 1.000 | 1.03 ± 0.03 | 0.352 |
|  | 3 | 5 | 0.60 ± 0.11 | 0.961 | 0.08 ± 0.02 | 1.000 | 1.00 ± 0.06 | 0.425 |
|  | 6 | 5 | 0.49 ± 0.08 | 0.972 | 0.13 ± 0.02 | 1.000 | 1.08 ± 0.06 | 0.114 |
|  |  |  | P=0.985 |  | P=0.911 |  | P=0.123 |  |
| **Female** |  |  |  |  |  |  |  |  |
| Sham Control | 0 | 5 | 0.62 ± 0.07 |  | 0.13 ± 0.04 |  | 0.66 ± 0.08 |  |
| CDMA | 1.5 | 5 | 0.54 ± 0.08 | 1.000 | 0.18 ± 0.03 | 0.263 | 0.92 ± 0.17 | 0.337 |
|  | 3 | 5 | 0.72 ± 0.12 | 0.778 | 0.16 ± 0.02 | 0.316 | 0.73 ± 0.12 | 0.406 |
|  | 6 | 5 | 0.51 ± 0.04 | 1.000 | 0.19 ± 0.06 | 0.219 | 0.82 ± 0.05 | 0.297 |
|  |  |  | P=0.541 |  | P=0.212 |  | P=0.430 |  |
| GSM | 1.5 | 5 | 0.61 ± 0.10 | 0.519 | 0.20 ± 0.04 | 0.377 | 0.76 ± 0.07 | 0.376 |
|  | 3 | 5 | 0.70 ± 0.08 | 0.495 | 0.11 ± 0.02 | 0.447 | 0.74 ± 0.09 | 0.455 |
|  | 6 | 5 | 0.59 ± 0.07 | 0.525 | 0.13 ± 0.03 | 0.476 | 0.99 ± 0.03 | 0.010 |
|  |  |  | P=0.566 |  | P=0.737 |  | P=0.008 |  |

a Exposure began *in utero* on gestation day 5; RET=reticulocytes (immature erythrocytes).

b Mean ± standard error

c Pairwise comparison with the sham control group; exposed group values are significant at P≤0.025 by Williams’ or Dunn’s test.

d Sham control; No exposure to CDMA- or GSM-modulated cell phone RFR

e Dose-related trend derived from one-tailed linear regression or Jonckheere’s test; the trend is significant when P≤0.025.

**Supporting Information Table II**

##### Frequency of Micronuclei in Peripheral Blood Erythrocytes of Mice Following Exposure to CDMA- or GSM-

**Modulated Cell Phone RFR for 14 Weeks**a

|  |  |  |  |  |  |  |  |  |
| --- | --- | --- | --- | --- | --- | --- | --- | --- |
|  | **Dose (W/kg)** | **Number of Rats with Erythrocytes Scored** | **Micronucleated RETs/**  **1,000 RETs**b | **P Value**c | **Micronucleated Mature Erythrocytes/**  **1,000 Mature Erythrocytes**b | **P Value**c | **RETs**b **(%)** | **P Value**c |
| **Male** |  |  |  |  |  |  |  |  |
| Sham Controld | 0 | 5 | 2.55 ± 0.11 |  | 1.50 ± 0.04 |  | 1.43 ± 0.04 |  |
| CDMA | 2.5 | 5 | 2.44 ± 0.13 | 0.611 | 1.45 ± 0.03 | 0.748 | 1.45 ± 0.04 | 0.765 |
|  | 5 | 5 | 2.77 ± 0.13 | 0.168 | 1.46 ± 0.04 | 0.827 | 1.48 ± 0.04 | 0.736 |
|  | 10 | 5 | 2.93 ± 0.18 | 0.044 | 1.49 ± 0.02 | 0.736 | 1.45 ± 0.04 | 0.778 |
|  |  |  | P=0.013e |  | P=0.497 |  | P=0.803 |  |
| GSM | 2.5 | 5 | 2.84 ± 0.14 | 0.384 | 1.49 ± 0.04 | 0.695 | 1.39 ± 0.04 | 0.667 |
|  | 5 | 5 | 2.47 ± 0.19 | 0.455 | 1.45 ± 0.02 | 0.781 | 1.38 ± 0.04 | 0.787 |
|  | 10 | 5 | 2.53 ± 0.13 | 0.484 | 1.50 ± 0.02 | 0.675 | 1.45 ± 0.07 | 0.830 |
|  |  |  | P=0.733 |  | P=0.561 |  | P=0.809 |  |
| **Female** |  |  |  |  |  |  |  |  |
| Sham Control | 0 | 5 | 2.72 ± 0.27 |  | 1.18 ± 0.02 |  | 1.31 ± 0.11 |  |
| CDMA | 2.5 | 5 | 2.16 ± 0.15 | 0.846 | 1.06 ± 0.04 | 0.956 | 1.31 ± 0.12 | 1.000 |
|  | 5 | 5 | 2.32 ± 0.22 | 0.908 | 1.09 ± 0.03 | 0.982 | 1.43 ± 0.11 | 0.930 |
|  | 10 | 5 | 2.48 ± 0.20 | 0.883 | 1.14 ± 0.02 | 0.929 | 1.26 ± 0.09 | 0.935 |
|  |  |  | P=0.629 |  | P=0.585 |  | P=0.843 |  |
| GSM | 2.5 | 5 | 2.50 ± 0.40 | 0.774 | 1.14 ± 0.05 | 0.827 | 1.18 ± 0.08 | 0.671 |
|  | 5 | 5 | 2.35 ± 0.15 | 0.850 | 1.09 ± 0.02 | 0.893 | 1.16 ± 0.06 | 0.791 |
|  | 10 | 5 | 2.16 ± 0.15 | 0.878 | 1.12 ± 0.04 | 0.916 | 1.43 ± 0.08 | 0.438 |
|  |  |  | P=0.937 |  | P=0.891 |  | P=0.245 |  |

a Mice were 5 to 6 weeks old when exposure began; RET=reticulocyte (immature erythrocyte)

b Mean ± standard error

c Pairwise comparison with the sham control group; exposed group values are significant at P≤0.025 by Williams’ test.

d Sham control; No exposure to CDMA- or GSM-modulated cell phone RFR

e Dose-related trend derived from one-tailed linear regression or Jonckheere’s test; the trend is significant when P≤0.025.
